# Comparative analysis of cellular immune responses to four seasonal inactivated influenza vaccines in younger and older adults

**DOI:** 10.1093/jimmun/vkaf286

**Published:** 2026-02-09

**Authors:** Vanessa Silva-Moraes, Laise Rodrigues Reis, Ted M. Ross

**Affiliations:** 1 Florida Research and Innovation Center, Cleveland Clinic, Port Saint Lucie, FL, United States; 2 Department of Infection Biology, Lerner Research Institute, Cleveland Clinic, Cleveland, OH, United States; 3 Center for Vaccines and Immunology, University of Georgia, Athens, GA, United States; 4 Department of Infectious Diseases, University of Georgia, Athens, GA, United States

**Keywords:** B cells, T cells, influenza, vaccination

## Abstract

The efficacy of seasonal influenza vaccines varies across age groups, even in individuals with strong antibody responses. Since protection against influenza virus infection also involves cellular immunity, identifying immune markers beyond neutralizing antibodies is crucial for informing the design of next-generation vaccines. Participants were categorized by age, 28–60 and 65–85 yr, and were vaccinated with 1 of 4 influenza vaccines (Fluzone Standard-Dose [FSD], Flucelvax [FCEL], Fluzone High-Dose [FHD], or Fluad [FAD]) during the 2023–2024 influenza season. Blood samples were collected before and after vaccination and analyzed for hemagglutination inhibition (HAI) activity, antibody-secreting cells (ASCs), and HA-specific compartments: memory B cells (MBCs), circulating T follicular helper (cTfh) cells, and CD4^+^ T effector cells. Although all vaccines increased HAI titers, FHD, which contains 4 times more antigen content than the other vaccines, elicited superior cellular responses compared to FAD in older participants. FCEL, produced in mammalian cells, was more effective than the egg-produced FSD in younger adults. Notably, FHD triggered simultaneous ASC and cTfh1 activation in older adults, which were linked to HA-specific MBCs and long-term humoral responses. FCEL induced early and cytokine-secreting HA-specific CD4^+^ T cell responses in younger adults, correlating with early B cell proliferation and enhanced antibody production. These findings highlight the critical role of antigen dose and quality in inducing HA-specific cellular immunity and coordinating B and T cell activation. Synergistically engaging ASCs, cTfh, MBCs, and CD4^+^ T cells to enhance immunological memory and establish long-term vaccine-induced immunity should be considered in future influenza vaccine design.

## Introduction

Influenza remains a major global health concern, causing significant morbidity and mortality, particularly among vulnerable populations such as the elderly, young children, and individuals with compromised immune systems.^1,2^ Vaccination is one of the primary tools for combating influenza virus infection and disease. However, existing vaccines have limitations regarding their effectiveness and the level of protection. Since influenza viruses evolve rapidly, this leads to a decrease in the effectiveness of current vaccines, particularly when the circulating strains are mismatched to the vaccine components.^2,3^

Current influenza vaccines primarily target the surface glycoprotein hemagglutinin (HA), since the elicitation of protective antibodies against HA is crucial to neutralize virus infection.^3^ Several commercial seasonal inactivated influenza vaccines (IIV) are on the market^4^ and containing H1N1, H3N2, and B strains. Fluzone Standard Dose (FSD), produced by Sanofi Pasteur is a split IIV and is the most commonly administered vaccine each season.^5^ This egg-based vaccine contains 15 mg of HA per strain, without the inclusion of an adjuvant.^6,7^ Flucelvax (FCEL) produced by Seqirus, is an IIV (15 mg of HA per strain) that can be administered to people with egg allergies due to its production in mammalian cells.^8^ Enhanced IIV options, designed for older adults (65+), include egg-based produced Fluzone High-Dose (FHD)^9–11^ and Fluad (FAD).^12^ FHD contains 60 mg of HA per strain with no adjuvant.^9–11^ FAD, made by Seqirus, contains 15 mg of HA per strain and is formulated with the MF59 squalene-based oil-in-water emulsion adjuvant.^12^ FAD boosts IFN-γ^+^ T cells^13^ and CD4^+^ T helper cell activity.^14^ Conversely, FHD stimulates greater T follicular helper (Tfh) cell activation and plasmablast recruitment,^15^ while IFN-γ^+^ T cell responses are comparable to those of a standard dose.^16^

A universal influenza virus vaccine that offers broad protection across different virus strains and age groups remains a key global priority. Most vaccine development efforts focus on enhancing hemagglutinin inhibition (HAI) titers to the HA glycoprotein, aiming to confer superior immunogenicity and vaccine efficacy.^17,18^ However, protection against influenza is not solely reliant on the humoral (antibody-mediated) response. High levels of HA-specific antibodies are associated with reduced risk and severity of infection, but they do not provide complete sterilizing immunity, and breakthrough infections can still occur.^19^ The immune response against influenza viruses also relies on other aspects of the immune system, particularly cellular immunity, which includes B cells and T cells.^20–25^ These different immune system components work together to establish a robust and lasting immune response, yet they remain underexplored. A comprehensive understanding of how currently available influenza vaccines stimulate both humoral and cellular immune responses could be useful for advancing vaccine development.

Recent advancements have highlighted the importance of immune markers beyond neutralizing antibodies. CD8^+^ and CD4^+^ T cells can reduce disease severity in the absence of neutralizing antibodies.^26^ Memory T cell responses are associated with protection against symptomatic infection and may serve as additional correlates of protection.^16,27^ Circulating Tfh (cTfh) cells are particularly important in generating antibody responses following IIV vaccination^28^ and both cTfh type-1 cells and antibody-secreting cells (ASCs) are associated to the development of memory B cells (MBCs) and long-lasting antibody production.^22–24^ However, the specific response targeting HA elicited from all the different immune compartments remains poorly explored. While responses to internal viral proteins are often dominant, due to their high conservation, activation of CD4^+^ T cells specific to surface HA are correlated with the development of HA-specific neutralizing antibodies, which play a vital role in protective immunity.^29–31^

In this study, longitudinal samples were collected from healthy individuals following vaccination with FSD and FCEL (younger adults) or FHD and FAD (older adults) to examine the interaction of different adaptive immune response components. The interplay between humoral and cellular immune components such as antibody, ASCs, MBCs, cTfh cells, CD4^+^ T cells, and cytokine-producing CD4^+^ T cells were examined. Overall, FHD induced the strongest immune responses in older adults, with acute immune markers closely linked to memory B and T cells, highlighting the long-lasting effects on immunity. In younger adults, FCEL induced the highest magnitude of effector HA-specific CD4^+^ T cells, which correlated with ASC and humoral immunity. By integrating a broad spectrum of immune parameters from different vaccine platforms, these findings provide deeper insights into both humoral and cellular immune responses, with implications for universal vaccine development.

## Material and methods

### Study population

**Figure 1.**
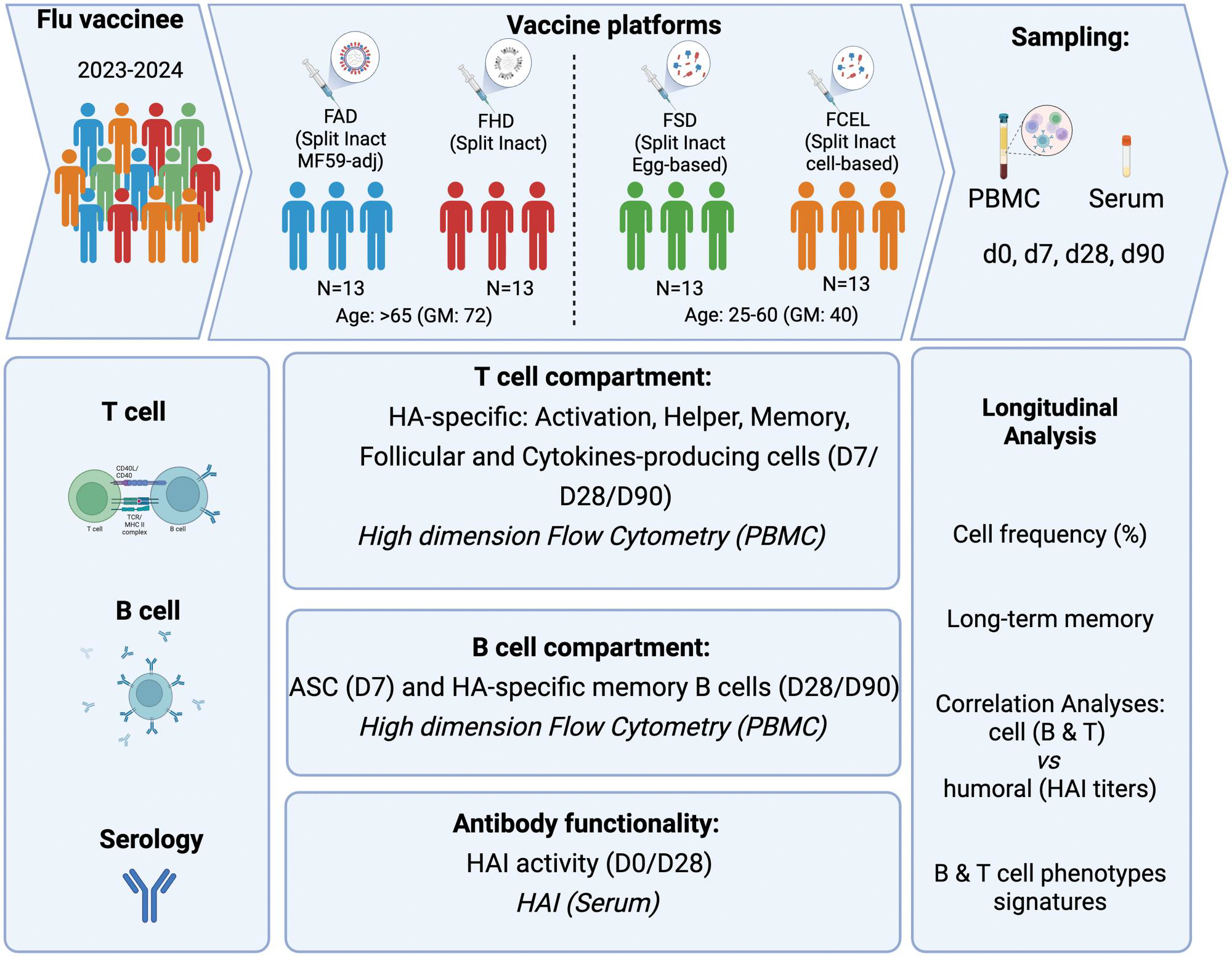
Experimental design. Cohort summary and methods design for analyzing H1N1 and H3N2 HA-specific humoral and cellular responses following vaccination with FAD, FHD, FSD, and FCEL. FAD, Fluad; FHD, Fluzone High-Dose; FSD, Fluzone Standard-Dose; FCEL, Flucelvax. Created with BioRender.com.

Participants were recruited between September 2023 and March 2024 in Athens, Georgia, and Port Saint Lucie, Florida, USA. All participants provided written informed consent, and the research protocol was approved by the University of Georgia (IRB no. 20224877) and the Cleveland Clinic Florida Institutional Review Boards (IRB no. 23–329). The study population comprised 52 healthy adults: 26 younger (ages 28–60) and 26 older (65–85) (Fig. 1, Table 1). Younger adults were either vaccinated with FSD (split-inactivated, Sanofi Pasteur, 15 μg HA per strain) or FCEL (cell-based, inactivated, Seqirus, 15 μg HA per strain). Older adults received either the FHD (60 μg HA per strain) or FAD (MF59-adjuvanted, Seqirus, 15 μg HA per strain). All vaccines were quadrivalent and contained antigens from all 4 strains: H1N1, H3N2, B Yamagata, and Victoria lineage. Fluzone SD and HD contained antigens from A/Victoria/4897/2022 (H1N1), A/Darwin/9/2021 (H3N2), B/Phuket/3073/2013 (Yamagata lineage), and B/Michigan/01/2021 (Victoria lineage). FAD was modified on A/Darwin/6/2021 IVR-227 (H3N2) and B/Austria/1359417/2021 BVR-26 (Victoria lineage). FCEL uses A/Georgia/12/2022 CVR-167 (H1N1), A/Darwin/11/2021 (H3N2), B/Singapore/WUH4618/2021 (Victoria lineage), and B/Singapore/INFTT-16–0610/2016 (Yamagata lineage). Vaccine-induced humoral and cellular responses to the H1N1 and H3N2 components of the influenza virus vaccine were evaluated.

### Sampling

Blood samples were collected from all participants before vaccination (day 0; D0), and 7 (D7), 28 (D28), and 90 d (D90) after vaccination. Samples were collected in vacutainer serum separation tubes (SST) (BD Biosciences, Franklin Lakes, New Jersey, USA) and centrifuged at 1,000 × *g* for 10 min at room temperature (RT). After centrifugation, the serum was isolated from the gel layer and red blood cell pellet. Additional samples were collected in vacutainer K2 EDTA tubes (BD Biosciences, Franklin Lakes, New Jersey, USA), and samples were homogenized and transferred to vacutainer cell preparation tubes (CPT) (BD Biosciences, Franklin Lakes, New Jersey, USA) for peripheral blood mononuclear cells (PBMC) isolation. The tubes were centrifuged at 1,800 × *g* for 20 min at RT. Sera were removed and PBMCs were washed in 1× phosphate-buffered saline (PBS; Corning, Manassas, Virginia, USA). Residual red blood cells were removed by incubation with ACK red blood cell (RBC) lysis buffer (Life Technologies, Carlsbad, California, USA). After additional washes, cells were resuspended in cold freezing media (90% Bovine Serum with 10% DMSO) to a final concentration of 1 × 10^7^ cells/ml. Cells were stored in liquid nitrogen until use for cellular analysis.

### Influenza hemagglutination-inhibition (HAI) assay

HAI assays were performed to quantify antibodies capable of inhibiting influenza virus binding to sialic acid on erythrocytes. The assay was conducted as previously described. Briefly, Receptor Destroying Enzyme (RDE; Denka Seiken, Co., Japan)-treated and heat-inactivated sera were added to 6 volumes of PBS and diluted 2-fold in V-bottom 96-well plates. The H1N1 (A/Victoria/4897/2022) and H3N2 (A/Darwin/9/2021) strains were adjusted to a concentration of 8 hemagglutination units (HAU)/50 μl, and an equal volume was added to the V-bottom microtiter plates. Plates were incubated for 20 min at RT followed by the addition of 0.8% turkey red blood cells (Lampire Biologicals, Pipersville, Pennsylvania, USA) for an additional 30 min. HAI titers were recorded as the reciprocal of the highest sera dilution that inhibited hemagglutination. Seroprotection was classified as HAI titers ≥1:40, while seroconversion was defined as a ≥ 4-fold increase in the ≥1:40 HAI titer.

### Detection of antibody-secreting cells (ASC) and circulating T follicular helper cells (cTfh)

ASCs (CD19^+^CD27^hi^CD38^hi^, plasmablasts, and plasma cells, collectively known as antibody-secreting cells) and cTfh cells were measured before (D0) and 7 days (D7) post-vaccination by flow cytometry. We quantified total cTfh as a surrogate for influenza virus-specific Tfh response occurring in the lymphoid compartment. PBMCs were thawed and resuspended in 10 ml of culture media (RPMI-1640 media containing 2 mM L-glutamine, sodium bicarbonate, and supplemented with 10% FBS, 1% penicillin/streptomycin, 2-ME, sodium pyruvate, and nonessential amino acids) in the presence of 50 U/ml of Benzonase (70664–3, Millipore, Burlington, Massachusetts, USA). Cells were centrifuged at 500×*g* for 10 min and then resuspended in media containing 100 μg/ml of DNase (DN25–1G, Sigma, St Louis, Missouri, USA). Cells were counted on a LUNA-IITM cell counter (Logos Biosystems, Korea) and rested in the same media at 2 × 10^6^ cells/ml for 3 h at 37 °C, 5% CO2. Next, cells were washed, resuspended in culture media without DNase, and approximately 3–4 × 10^6^ cells were plated in U-bottom 96-well plates. Cells were incubated with Fc block solution (2.5 μl/well, no. 564220, BD Biosciences, Franklin Lakes, New Jersey, USA) and LIVE/DEAD NIR marker (no. L10119, Thermo Scientific, Waltham, Massachusetts, USA) for 20 min at 4 °C. Cells were spun down and stained with 50 μl/well of surface antibodies for 30 min at 4 °C in the dark. After surface staining, cells were washed twice and then resuspended in FACS buffer (PBS with 0.5% BSA and 2 mM EDTA) for acquisition on the Aurora Spectral Flow Cytometer system with four lasers (Cytek Bioscience, Fremont, California, USA). All antibodies are listed in Table S1A. A representative gating strategy of ASCs and cTFh cells is shown in Fig. S1A.

### Detection of HA-specific memory B cells (MBC)

HA-specific MBCs were detected using biotinylated proteins combined with different streptavidin (SA) fluorophore conjugates, as previously described with modifications.^22,32^ Since the probe for A/Victoria/4897/2022 (H1N1) (2023–2024 vaccine strain) was not available, we employed A/Wisconsin/588/2019 (H1N1) (2022–2023 vaccine strain), an alternative probe previously used by our group.^20^ H3N2-specific B cells were detected using a probe matched to the vaccine strain (A/Darwin/6/2021). First, influenza HA recombinant antigens A/Wisconsin/588/2019 (H1N1) and A/Darwin/6/2021 (H3N2) were produced in-house and biotinylated using a BirA500: BirA biotin-protein ligase standard reaction kit (Avidity Biosciences, Inc, San Diego, California, USA), according to the manufacturer’s instructions. Excess biotin was subsequently removed using Amicon Ultra-15 Centrifugal Filter Units 30K MWCO (Thermo Scientific, Waltham, Massachusetts, USA), and the protein was quantified with a Pierce BCA Assay (Thermo Scientific, Waltham, Massachusetts, USA). Biotinylated proteins were individually multimerized with fluorescently labeled SA for 1 h at 4 °C at a 1:2.6 mass ratio (eg, 60 ng HA with 156 ng SA). HA (H1N1) was labeled with SA-PE (#405245, Biolegend, San Diego, California, USA), and HA (H3N2) was labeled with SA-APC (#405243, Biolegend, San Diego, California, USA). SA-APC/CY7 (#405208, Biolegend, San Diego, California, USA) was used as a decoy probe without biotinylated protein to gate out cells that bind non-specifically to streptavidin. For staining, cryopreserved PBMCs were thawed and rested as described in item 2.4, and 3–4 × 10^6^ cells were prepared in a 96-well U-bottom plate. Cells were first incubated with Fc block (2.5 μl/well, #564220, BD Biosciences, Franklin Lakes, New Jersey, USA) and stained with LIVE/DEAD NIR marker (#L10119, Thermo Scientific, Waltham, Massachusetts, USA) at 50 μl/well for 20 min at 4 °C. Cells were then washed and stained with a 50 μl antigen probe master mix containing 60 ng HA H1N1-PE, 60 ng HA H3N2-APC, and 10 ng SA-APC/CY7 decoy, prepared in FACS with 5 μM free D-biotin (Avidity Biosciences, Inc, San Diego, California, USA) for 1 h on ice at 4 °C. HA probes were prepared individually and mixed after multimerization with free D-biotin to minimize potential cross-reactivity between probes. Following incubation with the antigen probe, cells were washed 3 times with FACS buffer and stained with 50 μl/well of surface antibodies for 30 min at 4 °C in the dark. After surface staining, cells were washed twice and resuspended in FACS buffer for acquisition on an Aurora Spectral Flow Cytometer system with four lasers (Cytek Bioscience, Fremont, California, USA). All antibodies are listed in Table S1B. A representative gating strategy for HA-specific MBCs is shown in Fig. S1B. The positivity rate was classified as a ≥ 2-fold increase in baseline frequency (D0).

### T cell activation-induced markers (AIM) assay and intracellular cytokine staining (ICS)

Peptide pools (PepMix^™^) spanning the H1N1 and H3N2 HA sequences were obtained from JPT Technologies (Berlin, Germany). H1N1 HA PepMix^™^ (#PM-INFA-HAVIC19–1) was composed of a pool of 139 peptides derived from a peptide scan (15mers with 11 aa overlap) through hemagglutinin (Swiss-Prot ID: EPI_ISL_417210 [GISAID]) of influenza A, isolate A/Victoria/2570/2019 (H1N1). H3N2 HA PepMix^™^ (#PM-INFA-HADAR21–1) was composed of pool of 139 peptides derived from a peptide scan (15mers with 11 aa overlap) through hemagglutinin (Swiss-Prot ID: EPI_ISL_2233240 [GISAID]) of influenza A, isolate A/Darwin/9/2021 (H3N2). The peptides pool was resuspended in DMSO and used at 1 μg/ml final concentration.

The AIM assays and ICS were performed as previously described,^33,34^ with modifications. To define HA-specific T cells by AIM (AIM^+^ cells), CD4^+^ T cells were measured as a percentage of CD69^+^CD40L^+^ after peptide stimulation. The AIM assay also defined HA-specific cTfh and memory subsets (CD45RA, CCR7). To define the HA-specific T cells by the ICS assay, cytokine-producing cells were gated together with the expression of CD40L on CD4^+^ T cells. T cell analysis was performed in matched specimens across all 4 timepoints (D0, D7, D28, D90), unless otherwise stated.

PBMCs were thawed and rested as described in item 2.4. Next, the cells were washed, resuspended in culture media without DNAse and plated in U-bottom 96-well plates at a density of 1 × 10^6^ cells/well. Before adding the stimulus, PBMCs were blocked at 37 °C for 15 min with 0.5 μg/ml anti-CD40 mAb (#130–094-133, Miltenyi Biotec, Bergisch Gladbach, Germany). Subsequently, H1 and H3 HA-specific peptides were added in the presence of anti-CD28 (#302902, Biolegend, San Diego, California, USA) at 1 μg/ml and 1 μl/well of fluorescently labeled chemokine receptor antibodies (anti-CCR6, CXCR5, CXCR3, and CCR7). Additionally, PBMCs were incubated with an equimolar amount of DMSO as a negative control and with 5 mg/ml of phytohemagglutinin (PHA, Roche, Basel, Switzerland) as a positive control. After 4 h of incubation at 37 °C and 5% CO_2_, the protein transport inhibitor cocktail (#00–4980-03, Invitrogen, Waltham, Massachusetts, USA) was added for a further 20 h of incubation (for a total incubation of 24 h in a total volume of 200 μl). Following incubation, plates were centrifuged at 705×*g* for 10 min and incubated in PBS+EDTA 2 mM for 5 min at RT.

For the AIM stain, cells were washed and resuspended in 50 μl/well of PBS containing human Fc block (2.5 μl/well, #564220, BD Biosciences, Franklin Lakes, New Jersey, USA) and the LIVE/DEAD NIR marker (#L10119, Thermo Scientific, Waltham, Massachusetts, USA) and incubated for 20 min at RT. Cells were spun down, and 50 μl/well of surface antibody diluted in FACS buffer was added and incubated for 40 min at 4 °C. Following surface staining, cells were washed twice in FACS buffer and proceeded to the ICS. Cells were fixed and permeabilized following the CytoFix/CytoPerm kit (#554714, BD Biosciences, Franklin Lakes, New Jersey, USA) and cycles of 1,100×*g* centrifugation for 6 min. Subsequently, cells were stained with 50 μl/well of intracellular antibodies for 30 min at RT in the dark. Cells were washed twice, resuspended in FACS buffer, and acquired on the Aurora Spectral Flow Cytometer system with four lasers (Cytek Bioscience, Fremont, California, USA).

A list of antibodies used in the AIM and ICS panel can be found in Table S1C, D. A representative gating strategy of HA-specific CD4^+^ T cells using the AIM assay is shown in Fig. S1C and of HA-specific CD4^+^ T cells producing IFN-γ, TNF-α, IL-2 using the ICS assay is shown in Fig. S1D. HA-specific CD4^+^ T cells were measured as background (DMSO) subtracted data, with a minimal DMSO level set to 0.002%. The limit of quantification (LOQ) was calculated using the mean plus two standard deviations of all negative controls. All samples were acquired on an Aurora Spectral Flow Cytometer system with 4 lasers (Cytek Bioscience, Fremont, California, USA).

### Statistical analysis

Flow cytometry data was analyzed using FlowJo 10.10. Statistical analyses were performed using GraphPad Prism 10 (GraphPad Software, San Diego, California, USA). Data are plotted as the median and interquartile range (IQR) unless otherwise stated. Mann–Whitney *U* or Wilcoxon tests were applied for unpaired or paired comparisons, respectively. Differences among 3 or more groups were evaluated using the Kruskal–Wallis test and Dunn’s post hoc test for multiple comparisons. The χ^2^ test determined the positivity rate comparison, and Spearman test the correlation coefficient. Details about significance are provided in the respective figure legends. Phenotypic and functional biomarker signatures were assessed by converting continuous variables into categorical data using the global median of each biomarker as the cutoff. Biomarkers with more than 50% of participants above the cutoff were underscored and selected to construct Venn diagram (https://bioinformatics.psb.ugent.be/webtools/Venn) and identify common biomarkers between vaccines (FAD × FHD; FSD × FCEL) and timepoints (D7 and D28 post-vaccination).

## Results

### Population

Humoral and cellular responses were examined following FAD, FHD, FSD, and FCEL vaccination (*n* = 13 per group) in 52 healthy adults aged 25 to 85, recruited from September 2023 to March 2024. Older adults received FAD and FHD, while younger adults received FSD and FCEL (Table 1). Participants were selected based on sample availability, matched specimens, and complete time-point collection, excluding any self-reported chronic or immunosuppressive medical conditions. For most participants, serological and cellular assays were performed in parallel when matched specimens and complete time points were available. There were no significant differences in age (older adults: ~72 years, younger adults: ~42 years) or ethnicity (older adults: ~95% white, younger adults: ~85% white) among participants. The majority of individuals were female (older adults: ~65% female, younger adults: ~85% female), with FCEL having the highest overall percentage at 92%.

### HAI titers elicited by one of four different vaccines in younger and older adults

**Figure 2.**
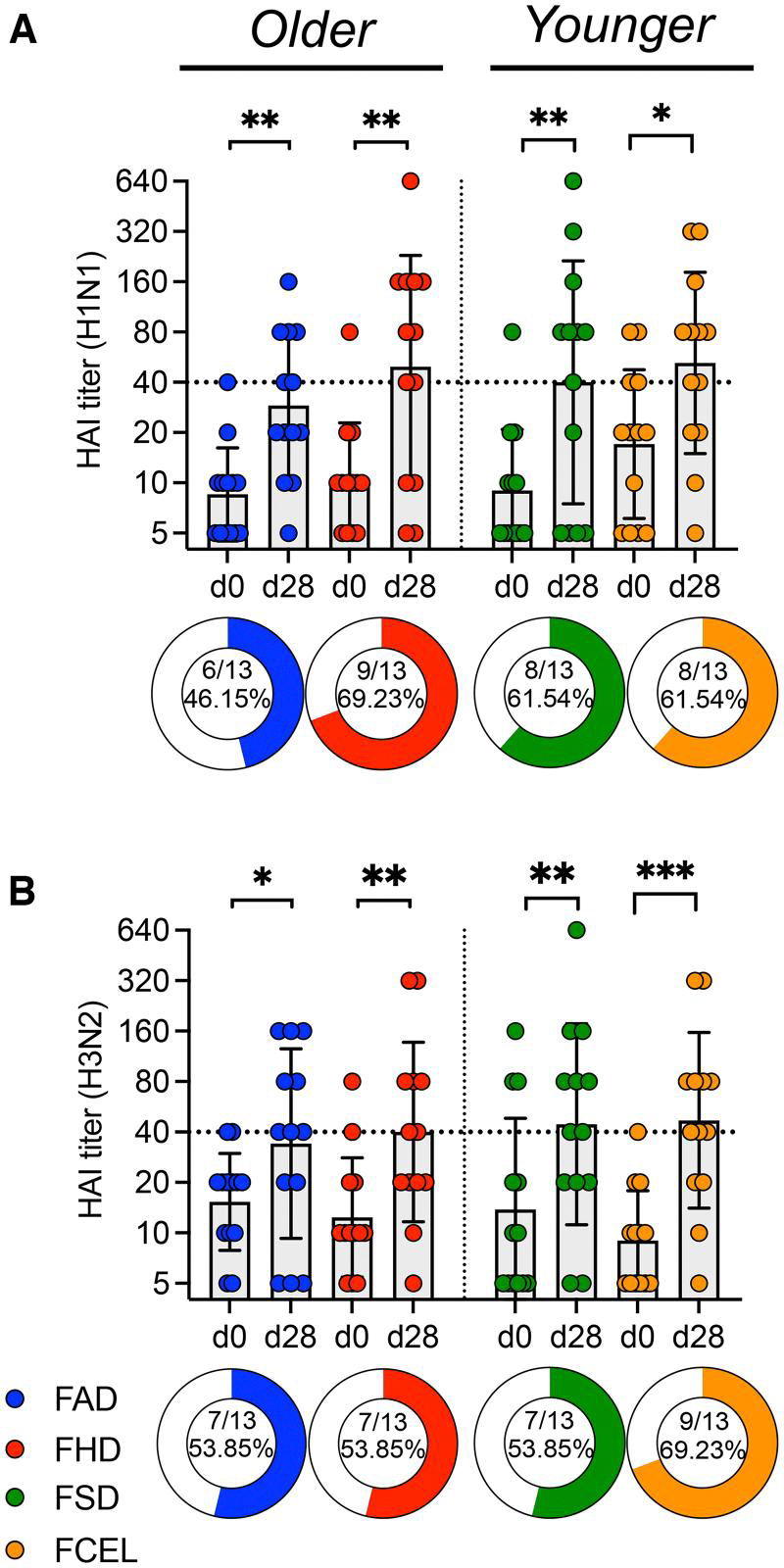
H1N1 and H3N2 HAI titers upon vaccination with FAD, FHD, FSD, and FCEL. H1N1 (A) and H3N2 (B) serum HAI titers were measured from the 2023–2024 season at day 0 (d0) and again 28 days (d28) after vaccination (*n* = 13 per vaccine group). The dotted black line indicates the seroprotective HAI titer of 40. The vaccine groups are color-coded. Data are represented as the geometric mean ± SD. Donut charts illustrate the proportion of participants exhibiting a detectable response (4-fold HAI increase and a seroprotective titer of ≥1:40). Data were analyzed for statistical significance using the Wilcoxon test (within the group) and the Mann–Whitney test (between different groups). Only significant data are stated (*P < 0.05; **P < 0.01; ***P < 0.001; ****P < 0.0001). FAD, Fluad; FHD, Fluzone High-Dose; FSD, Fluzone Standard-Dose; FCEL, Flucelvax; HAI, Hemagglutination-inhibition Assay.

HAI serum activity was assessed for all 52 donors before and 28 d after vaccination. All vaccine groups had a significant (*P* < 0.05) increase in the average HAI titer post-vaccination against the H1N1 influenza vaccine component (Fig. 2A) and H3N2 influenza vaccine component (Fig. 2B). Responders (ie, ≥4-fold increase in HAI titers ≥1:40) had a greater fold change for the H1N1 component (median = 8) compared to H3N2 component (median = 4) (median fold: H1N1 FAD = 8, FHD = 8, FSD = 8, FCEL = 4; H3N2 FAD = 4, FHD = 4, FSD = 4, FCEL = 8).

Older participants vaccinated with FHD had a higher positivity rate (≥4-fold increase in HAI titers ≥1:40) than FAD against H1N1 (69%) influenza virus 28 days post-vaccination. In younger participants, the FCEL group exhibited the highest positivity rate, with H1N1 at 62% and H3N2 at 69%. Additionally, participants vaccinated with FHD and FCEL had the highest magnitude response against H1N1 (geomean FAD = 29, FHD = 49, FSD = 40, FCEL = 52) and H3N2 (geomean FAD = 34, FHD = 44, FSD = 40, FCEL = 47) components following vaccination.

### cTfh-1 emerges concurrently with ASC and is associated with serological responses in FHD recipients

**Figure 3.**
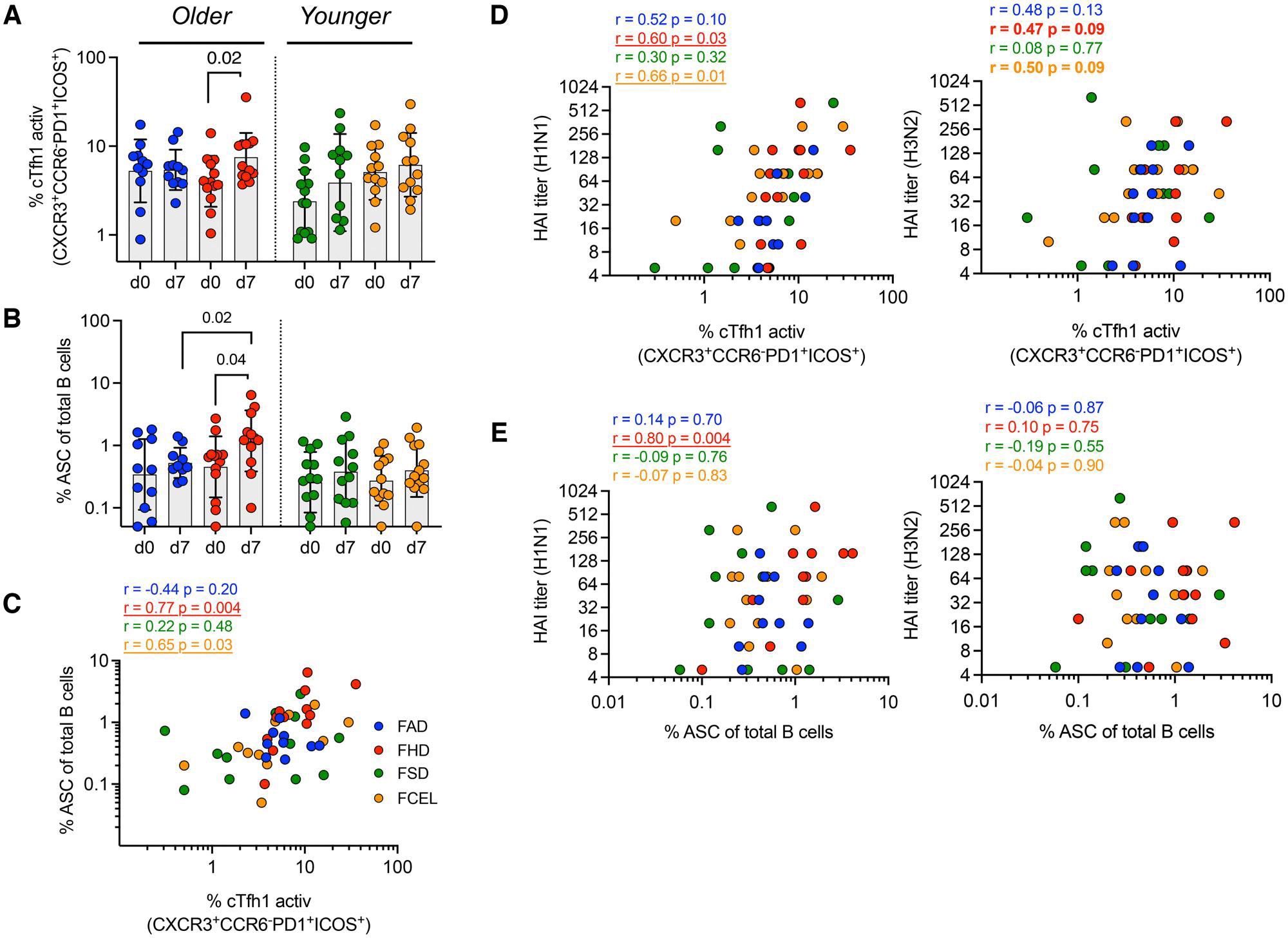
Early induction of cTfh and ASC in individuals upon vaccination with FAD, FHD, FSD and FCEL. The frequency of (A) activated circulating Tfh1 (CD4^+^CXCR5^+^CXCR3^+^CCR6^−^PD1^+^ICOS^+^) and (B) ASC (CD19^+^CD27^hi^CD38^hi^) was measured on day 0 (d0) and 7 days (d7) after vaccination (*n* = 13 per group). (C) The correlation between the frequency of cTfh1 and ASC on day 7. (D) The correlation between the frequency of cTfh on day 7 and H1N1 (left) and H3N2 (right) titers on day 28. (E) The correlation between the frequency of ASC on day 7 and H1N1 (left) and H3N2 (right) titers on day 28. The vaccine groups are color-coded. The data are represented as median and interquartile range (IQR). Data were analyzed for statistical significance using (A, B) Wilcoxon (within the group), Mann–Whitney (between different groups), and (C–E) Spearman’s (correlation). (A, B) Only significant data were represented, as indicated in the graphs. (C–E) Significant data are underlined, and trends are in bold. See Fig. S1A for the complete gating strategy. ASC, antibody-secreting cells; cTfh1, type-1 circulating T follicular helper; HAI, Hemagglutination-inhibition Assay; FAD, Fluad; FHD, Fluzone High-Dose; FSD, Fluzone Standard-Dose; FCEL, Flucelvax.

Participants vaccinated with FHD had a significantly higher number of activated cTfh1 cells (CXCR3^+^CCR6^−^PD1^+^ ICOS^+^) 7 d post-vaccination (*P* = 0.02) (Fig. 3A), with a greater fold-increase compared to participants vaccinated with FAD (*P* = 0.03) (Fig. S2). Simultaneously, there was an increase in ASCs in participants vaccinated with FHD (*P* = 0.04) 7 d after vaccination, which had a higher peak in ASCs compared to FAD-vaccinated participants (*P* = 0.02) (Fig. 3B).

To determine the role of activated cTfh1 in generating high-affinity memory B cells and inducing an early antibody response, the frequency of cTfh1 cells and ASCs at day 7 post-vaccination was correlated with HAI titers at day 28 post-vaccination. Participants vaccinated with FHD had early recruitment of ASCs and cTfh1 cells, as indicated by the positive and significant correlation observed at 7 d post-vaccination (Fig. 3C). At day 28 post-vaccination, there was a significant positive correlation between HAI titers specific for the H1N1 vaccine component and cTfh1 cells (*P* < 0.05), with similar trends between HAI titers specific for the H3N2 vaccine component and cTfh1 cells (*P* < 0.10) (Fig. 3D). There was a significant correlation between HAI iters at day 28 and acute ASCs isolated at 7 d post-vaccination in FHD participants against the H1N1 influenza virus component (Fig. 3E). Overall, FHD induced concurrent changes in the circulating B cell and Tfh cell compartments that were linked to the sustained serological responses observed after vaccination.

### HA-specific MBC induced by FHD and FCEL is associated with early effector cells

**Figure 4.**
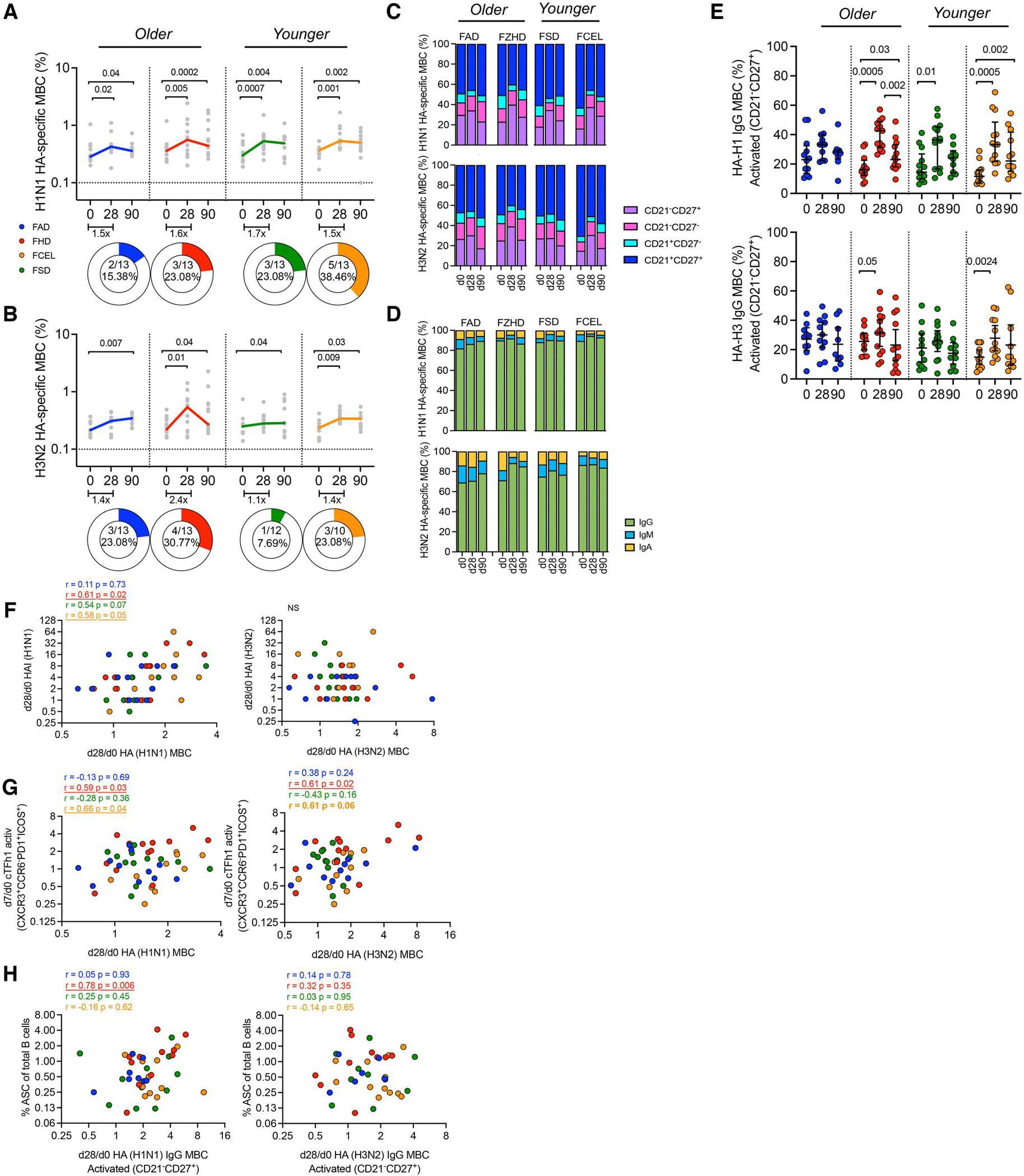
HA-specific MBC induced by FAD, FHD, FSD, and FCEL vaccination. The frequency of HA-specific (A) H1N1 and (B) H3N2 MBC (CD19^+^IgD^neg^) was measured before vaccination (d0) and day 28 and 90 post-vaccination (*n* = 13 per group) using designed HA-specific probes. The vaccines are color-coded, with the bold lines representing the median at each time point post-vaccination. Donut charts illustrate the proportion of participants showing a 2-fold baseline increase. The phenotype (C) and isotype (D) distributions of HA-specific MBC are depicted (top: H1N1, bottom: H3N2) at baseline (d0) and at d28 and d90 post-vaccination. Columns indicate the median frequency of MBC. (E) The frequencies of H1N1 and H3N2 IgG+ activated MBC (CD21^−^CD27^+^) were assessed before vaccination (d0) and at 28- and 90-days post-vaccination. The vaccine groups are color-coded. The data are represented as median and interquartile range (IQR). (F–H) Correlation analysis was conducted between the fold change of HA-MBC at d28 and (F) HAI titers at d28, (G) cTfh1 at d7, and (H) ASC at d7. Data were analyzed for statistical significance using (A, B, E) Wilcoxon (within the group), Mann–Whitney (between groups), (C, D) Two-way Tukey’s multiple comparison test, along with (F–H) Spearman’s correlation. Colored values refer to the group legend within the graph. Significant data are underlined, while trending data are bolded. See Fig. S1B for the full gating strategy. MBC, memory B cells; ASC, antibody-secreting cells; cTfh1, type-1 circulating T follicular helper cells; HAI, Hemagglutination-inhibition Assay; FAD, Fluad; FHD, Fluzone High-Dose; FSD, Fluzone Standard-Dose; FCEL, Flucelvax.

Recombinant HA probes corresponding to closely related HA proteins in the 2023–2024 vaccine strains were used to identify, quantify, and phenotypically characterize MBCs in vaccinated participants at 28 and 90 d post-immunization (Fig. 4). Even though HA probes were not the exact sequence matches to the vaccine strains, they allowed for a reliable assessment of HA-specific responses. Influenza HA-specific MBCs were identified among mature class-switched B cells (CD19^+^CD3^−^CD14^−^CD16^−^CD10^−^IgD^−^), excluding cells that bound free fluorochrome-streptavidin.

Participants vaccinated with any of the four influenza virus vaccines had significant increases (*P* < 0.05) in the frequency of H1 HA-specific MBC 28 d post-vaccination (FAD *P* = 0.02 1.5-fold, FHD *P* = 0.005 1.6-fold, FSD *P* = 0.0007 1.7-fold, FCEL *P* = 0.001 1.5-fold) (Fig. 4A). Additionally, participants vaccinated with FHD and FCEL had significant increases in MBC specific for H3 HA (FHD *P* = 0.01 2.4-fold, FCEL *P* = 0.009 1.4-fold) (Fig. 4B). The frequency of H1 and H3 MBCs in all vaccine groups decreased after 28 d. However, at 90 d post-vaccination, it remained significantly higher than the baseline levels at day 0 (*P* < 0.05). Both adult and elderly participants had statistically similar frequencies of H1 and H3 HA-specific MBCs at days 28 and 90 post-vaccination when vaccines were compared within of each age category (*P* > 0.05) (Fig. 4A, B). Overall, the FHD and FCEL groups had the highest positivity rates for both H1 (FHD 23%, FCEL 38%) (Fig. 4A) and H3 (FHD 31%, FCEL 23%) (Fig. 4B).

Activation (CD21) and memory (CD27) markers were analyzed on HA-specific MBCs pre- and post-vaccination (Fig. 4C). Participants vaccinated with FHD, FSD, or FCEL vaccines had significant increases in H1-HA-specific activated MBCs (CD21^−^CD27^+^) 28 d post-vaccination (FHD *P* < 0.0001, FSD *P* = 0.0007, FCEL *P* = 0.0004), while no significant changes were observed for participants vaccinated with FAD (*P* = 0.75) (Fig. 4C, top panel). H3-HA-specific activated MBCs increased significantly in FHD (*P* = 0.05) and FCEL (*P* = 0.02) (Fig. 4C, bottom panel). Although no significant differences were noted in the proportion of activated MBCs among participants vaccinated with any of the 4 influenza vaccines at 28 d post-vaccination, those vaccinated with FHD had the highest frequencies of H1- and H3-specific MBCs (~40%) in comparison to the other vaccine groups (Fig. 4C, top panel). Vaccinated participants had a lower proportion of resting MBCs (CD21^+^CD27^+^) at 28 d post-vaccination, with a significant decrease of H1-specific MBCs in participants vaccinated with FHD (*P* = 0.001) or FCEL (*P* = 0.006) (Fig. 4C, top panel). By day 90, activated MBCs decreased, becoming more resting and returning to their baseline state. The frequency of atypical memory B cells (CD21^−^CD27^−^) remained unchanged post-vaccination and was comparable among all participant groups (Fig. 4C).

H1 and H3 HA-specific MBCs had similar Ig isotype frequencies following vaccination in all participants regardless of the vaccine administered (Fig. 4D). At day 28 post-vaccination, there were slight increases in the frequency of IgG H1- and H3-HA MBCs, with lower frequencies of IgA and IgM, regardless of the vaccine administered. MBCs from participants vaccinated with FHD, FSD, and FCEL predominantly expressed IgG (H1 ~95% and H3 ~83%). Participants vaccinated with FAD had lower frequencies of both H1 (85%) and H3 (70%) IgG HA-specific MBCs (Fig. 4D).

Vaccination induced the expansion of activated HA-specific MBCs and a predominant IgG isotype. Phenotypic analysis of HA-specific IgG MBCs showed an activated phenotype that peaked 28 d post-vaccination (Fig. 4E). Participants vaccinated with FHD or FCEL had significant increases in activated HA-specific IgG^+^ MBCs against H1 (FHD and FCEL: *P* = 0.0005) and H3 vaccine components (FHD: *P* = 0.05 and FCEL: *P* = 0.002) at day 28 post-vaccination (Fig. 4E). Furthermore, FHD and FCEL recipients maintained a higher H1-specific response at D90 compared to baseline (D0, *P* < 0.05). At the same time, the FSD or FAD response transitioned to a predominantly resting phenotype, returning to baseline levels 90 d after vaccination.

The magnitude and rise of HA-specific MBCs post-vaccination were investigated in relation to the concurrent emergence of ASCs and cTfh1 cells on day 7, as well as the serological responses on day 28. The increase in H1-HA-specific MBCs at day 28 after vaccination was significantly correlated with the rise in HAI titers at day 28 post-vaccination (Fig. 4F) and with the early day 7 increase of cTfh1 in FHD and FCEL recipients (Fig. 4G). In particular, the rise of activated (CD21^−^CD27^+^) HA-specific IgG MBCs in FHD recipients was associated with the frequency of ASCs, which emerged as early as 7 d post-vaccination (*r*_s_ 0.78, *P* = 0.006) (Fig. 4H). There was no association between H3-HA-specific MBCs and serological responses or cellular events at day 7 post-vaccination. Overall, among all 4 vaccines, FHD and FCEL induced a significant expansion of HA-specific MBCs, which were predominantly IgG-activated and peaked at day 28 post-vaccination. Notably, participants vaccinated with FHD had MBCs linked with the emergence of acute key effector cells (cTfh1 cells and ASCs) and increased serological responses.

### Early recruitment of cytokine-secreting HA-specific CD4^+^ T cell response is associated with an increase in MBCs and long-term serological responses

**Figure 5.**
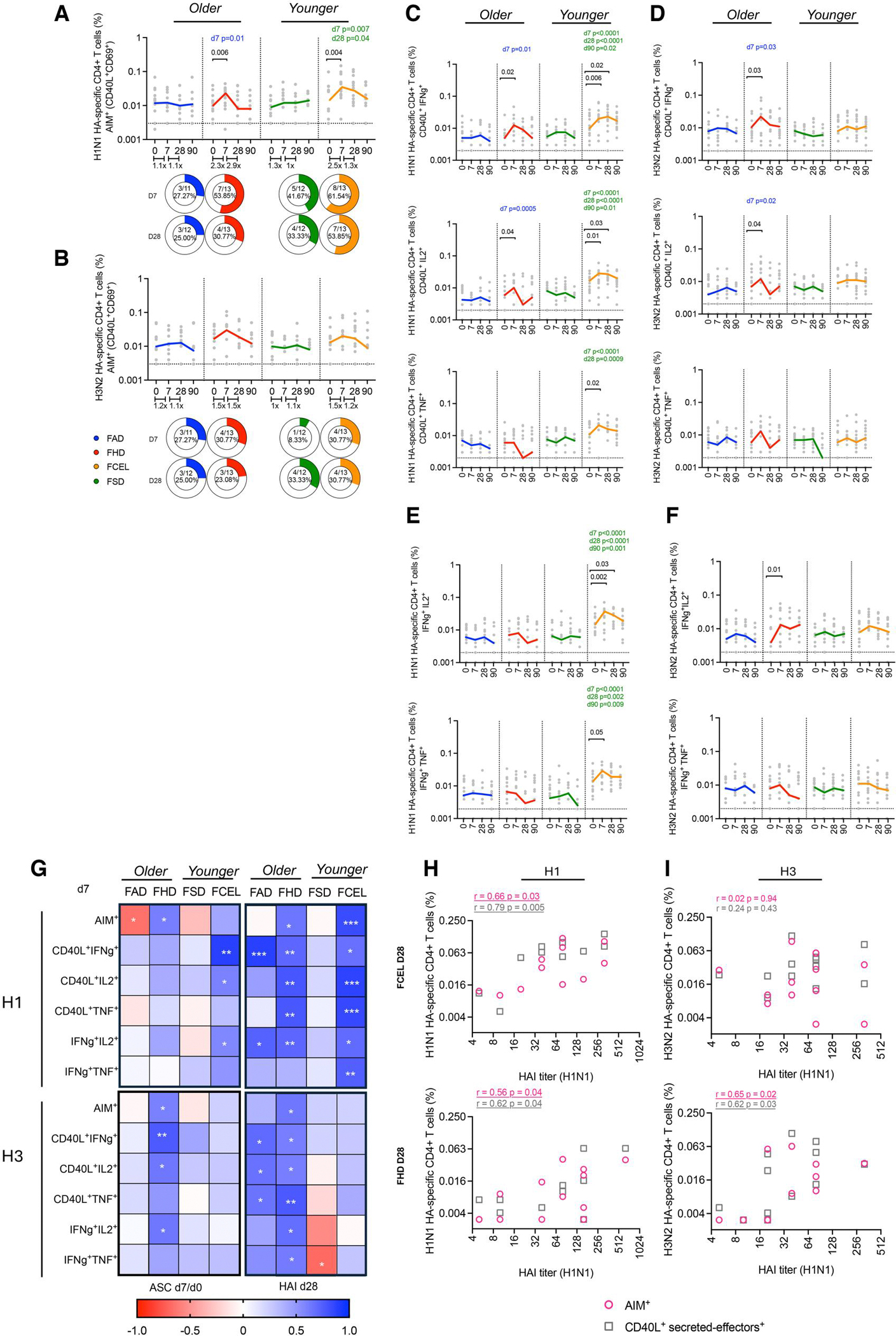
Longitudinal HA-specific CD4^+^ T cell responses measured by AIM and ICS assays following FAD, FHD, FSD, and FCEL vaccination. HA-specific CD4^+^ T cells were quantified as a percentage of AIM^+^ (CD40L^+^CD69^+^) CD4^+^ T cells after stimulating PBMCs with a peptide pool spanning the HA protein from (A) H1N1 and (B) H3N2. HA-specific functional CD4^+^ T cells were quantified as a percentage of CD40L^+^ secreted-effector cells producing IFN-γ, TNF-α, and IL-2 specific to (C) H1N1 and (D) H3N2 and as a percentage of double-positive secreting cells producing IFN-γ/TNF-α and IFN-γ/IL-2 specific to (E) H1N1 and (F) H3N2. The vaccines are color-coded, with the bold lines representing the median at each time point post-vaccination. Data are presented as background-subtracted values paired with unstimulated samples. The dotted black lines indicate the limit of quantification. (G) Correlation matrixes between the fold change of ASC at day 7 (left) and HAI at day 28 (right) across AIM^+^ and ICS^+^ metrics at day 7. (H, I) Correlation analysis between the frequency of AIM^+^ and ICS^+^ HA-specific CD4^+^ T cells at day 7 and HAI titers at day 28 induced by FHD and FCEL recipients. Data were analyzed for statistical significance using (A–F) Wilcoxon (within the group), Mann–Whitney (between groups), and (G–I) Spearman’s correlation. Colored values refer to the group legend within the graph. Significant data are underlined, while trending data are bolded. See Fig. S1C for the complete gating strategy. ASC, antibody-secreting cells; AIM, activation-induced marker; ICS, intracellular cytokine staining; HAI, Hemagglutination-inhibition Assay; FAD, Fluad; FHD, Fluzone High-Dose; FSD, Fluzone Standard-Dose; FCEL, Flucelvax.

To determine the response of T cells directed at the HA portion (other than total cTfh) following all 4 vaccines, HA-specific CD4^+^ T cells were assessed using the AIM assay (CD69^+^CD40L^+^) and the ICS assay (CD40L^+^ secreting effectors). Participants vaccinated with FHD (*P* = 0.006) and FCEL (*P* = 0.004) vaccines had significantly expanded H1N1-HA-specific T cells 7 d post-vaccination, with a positivity rate exceeding 50% (FHD 54%, FCEL 62%) (Fig. 5A). In FHD participants, these responses gradually declined (2.9-fold reduction, *P* = 0.08) by day 28, returning to baseline levels in both FHD and FCEL groups by day 90. In elderly participants, the FHD vaccine induced a greater magnitude of H1 AIM^+^ T cells (D7) than those vaccinated with FAD (*P* = 0.01). In younger participants, FCEL induced a higher frequency of H1 AIM^+^ T cells at day 7 (*P* = 0.007) compared to FSD, and these levels were maintained up to 28 d post-vaccination (*P* = 0.04) (Fig. 5A). Regardless of which of the 4 vaccines was administered, participants experienced a slight increase (~1.2-fold) in H3 HA-specific AIM^+^ T cells, with FHD and FCEL vaccinated participants showing the highest fold increase (1.5-fold) (Fig. 5B).

The ICS assay was used to investigate the functional profile of HA-specific T cells by measuring the frequencies of individual CD40L^+^ secreted-effector CD4^+^ T cells (IFN-γ^+^, TNF-α^+,^ and IL-2^+^) (Fig. 5C, D) and double-positive cytokine-secreting CD4^+^ T cells (IFN-γ^+^IL-2^+^ and IFN-γ^+^TNF-α^+^) following vaccination (Fig. 5E, F). Participants vaccinated with either FHD or FCEL vaccines had a significant peak in H1-HA-specific IFN-γ^+^ and IL-2^+^ T cells at day 7 post-vaccination (Fig. 5C). In FCEL participants, a significant increase in the frequency of double-cytokine-secreting CD4^+^ T cells was also observed (Fig. 5E). The increase in the frequency of cytokine-secreting CD4^+^ T cells specific to the HA-H3 component post vaccination was seen only in FHD recipients with a significant expansion of single CD40L^+^ secreted-effector IFN-γ^+^ and IL-2^+^ cells along with their double-secreting populations (IFN-γ^+^IL-2^+^ CD4^+^T cells) (Fig. 5D, F).

The T cell response (AIM and ICS) induced by FCEL and FHD vaccines demonstrated the strongest association across ASC and HAI responses at both early (D7) (Fig. 5G) and memory (D28) (Fig. 5H, I) stages. The frequency of H1 HA-specific CD4^+^ T cells induced by FHD and FCEL on day 7 significantly correlates with the increase in ASC at that same time point (Fig. 5G, left top panel). AIM^+^ and ICS^+^ CD4^+^ T cells induced by the same vaccines at the acute phase (D7) (Fig. 5G top right panel) and the memory phase (D28) (Fig. 5H) correlated with HAI titers 28 d post-vaccination. Additionally, the increase of AIM^+^ and ICS^+^ CD4^+^ T cells from FHD recipients was significantly associated with the increase of MBCs on day 28 (Fig. S3). Concerning the HA-H3-specific AIM^+^ and ICS^+^ CD4^+^ T cell response, only FHD recipients showed a significant association among these same metrics (ASC, HAI, and MBCs) (Fig. 5G bottom panel, 5I, S3). Overall, FCEL and FHD vaccines recruit early CD4^+^ T effector responses towards the HA antigen, which emerge simultaneously with ASC. These responses, particularly from FHD recipients, are linked to the rise of MBCs and increased HAI titers at a later stage (D28).

### HA-specific cTfh induced by FCEL and FHD is associated with ASC and late antibody responses

**Figure 6.**
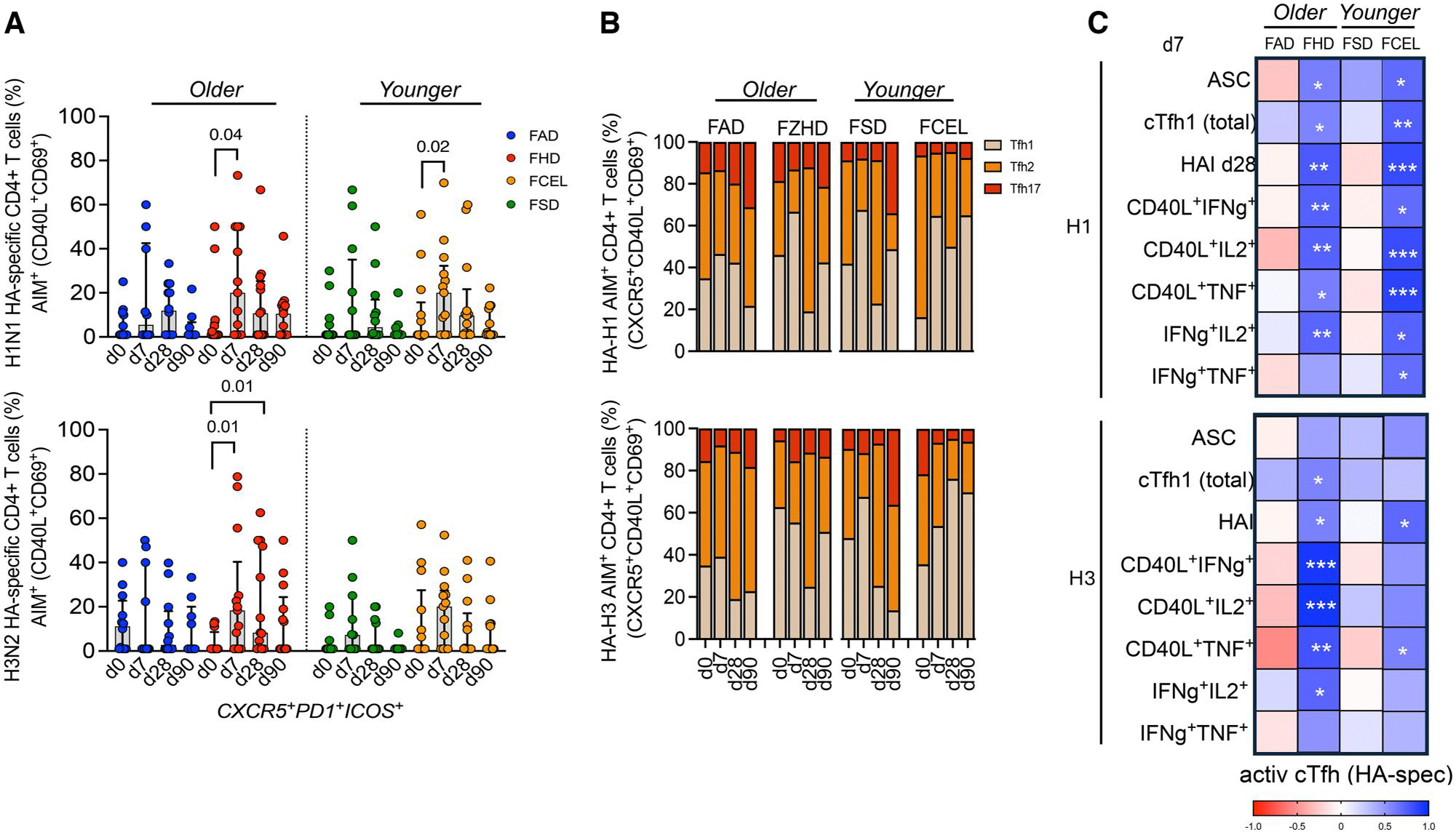
HA-specific cTfh responses measured by AIM following the FAD, FHD, FSD, and FCEL vaccination. (A) HA-specific CD4^+^ T cells were quantified as a percentage of AIM^+^ (CD40L^+^CD69^+^) CD4^+^ T cells after stimulating PBMCs with a peptide pool spanning the HA protein from H1N1 and H3N2. The vaccine groups are color-coded. The data are represented as median and interquartile range (IQR). Data are presented as background-subtracted values paired with unstimulated samples. (B) The phenotype distributions of HA-specific cTfh are depicted (top: H1N1, bottom: H3N2) at baseline (d0) and at days 7, 28, and 90 post-vaccinations. Columns indicate the median frequency of cTfh1–2-17. (C) Correlation matrixes between the activated cTfh (AIM^+^CXCR5^+^PD1^+^ICOS^+^) at day 7 across multiple parameters: ASC, total cTfh, and ICS^+^ CD4^+^ T cells at day 7 and HAI titers at day 28. Data were analyzed for statistical significance using (A) Mann–Whitney, (B) Two-way Tukey’s multiple comparison test, and (C) Spearman’s correlation. See Fig. S1C for the complete gating strategy. ASC, antibody-secreting cells; AIM, activation-induced marker; ICS, intracellular cytokine staining; HAI, Hemagglutination-inhibition Assay; FAD, Fluad; FHD, Fluzone High-Dose; FSD, Fluzone Standard-Dose; FCEL, Flucelvax.

To determine the specific response of cTfh cells against H1 and H3 HA proteins, HA-specific cTfh CD4^+^ T cells were designated as a subpopulation of AIM^+^ T cells. Participants vaccinated with FCEL and FHD had a significant expansion (*P* < 0.05) of activated HA-specific cTfh cells (AIM^+^CXCR5^+^PD1^+^ICOS^+^) against the H1 and H3 vaccine components at 7 d post-vaccination (Fig. 6A). Regardless of the vaccine used, participants predominantly had HA-specific cTfh1 cells (AIM^+^CXCR5^+^CXCR3^+^CCR6^−^) that peaked at day 7 post-vaccination. At day 28 post-vaccination, a higher frequency of cTfh2 was observed (Fig. 6B). Activated H1 HA-specific cTfh cells from FHD and FCEL recipients were correlated with ASCs and effector-secreted CD4^+^ T cells at day 7 post-vaccination, as well as correlated with H1N1-specific HAI titers at day 28 post-vaccination (Fig. 6C, top panel). Conversely, H3 HA-specific cTfh cells had a stronger association between the same metrics in FHD recipients compared to FCEL (Fig. 6C, bottom panel). Thus, participants vaccinated with FCEL and FHD displayed cTfh cells and cytokine-secreting CD4^+^ T cells specifically targeting the HA antigen, which expanded simultaneously with ASCs and are linked to humoral responses.

### Phenotypic and functional biomarker signatures in younger and older adults following vaccination

**Figure 7.**
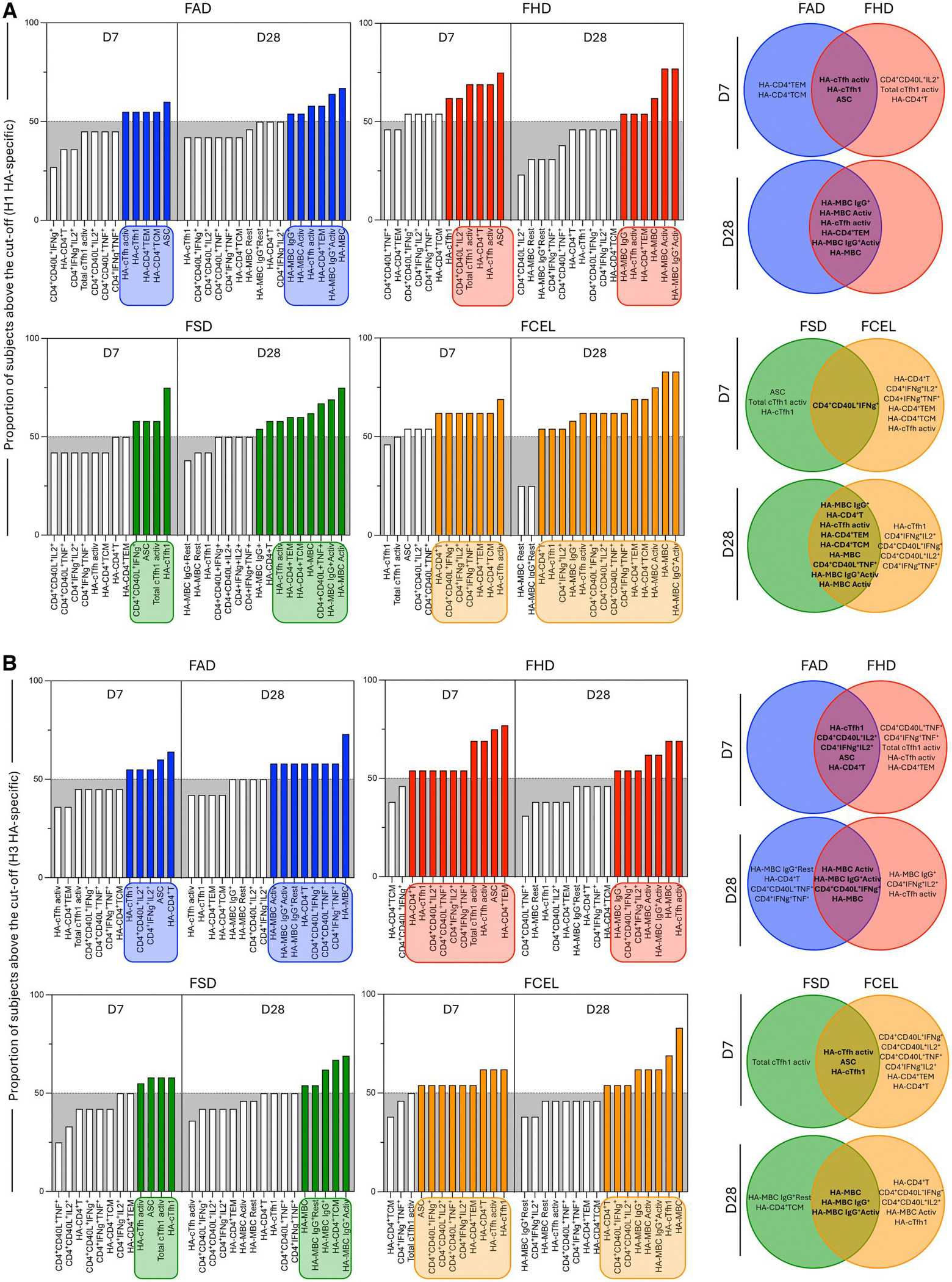
Phenotypic and functional biomarker signatures in younger and older adults following FAD, FHD, FSD and FCEL vaccination. Phenotypic and functional biomarker signatures of H1 (A) and H3 (B) HA-specific T- and B-cells were assessed at D7 and D28 post-vaccination in younger and older adults. Data analysis was carried out by converting continuous variables (percentage of gated cells) into categorical data using the global median of each biomarker as the cutoff. The proportion of subjects was assembled in ascending bar plots. Biomarkers with more than 50% of participants above the cutoff (gray area) were underscored (colored bars and rectangular frames) and selected to construct Venn diagram. Venn diagrams summarize common and unique biomarkers across vaccine groups and timepoints.

Phenotypic and functional biomarker signatures of H1 and H3 HA-specific T and B cells were profiled at D7 and D28 post-vaccination to provide an overview of the immune response in younger and older adults (Fig. 7). At D7 post-vaccination, data analysis revealed that FHD induced a broader and more cytokine-secreting immune response compared to FAD (Fig. 7A, B). Individuals vaccinated with FHD presented high levels of activated cTfh1 and H1 HA-specific secreting CD4^+^CD40L^+^IL2^+^ and HA-specific CD4^+^ T cells. Additionally, increased levels of H3 HA-specific TNF-α- and IFN-γ-secreting cells (CD4^+^CD40L^+^TNF-α^+^, CD4^+^IFN-γ^+^TNF-α^+^) and CD4^+^ T effector memory (HA-CD4^+^TEM) were also observed. In contrast, FAD elicited a more restricted immune profile, primarily including H1-specific CD4^+^ T effector and central memory (HA-CD4^+^TCM). A Venn diagram further illustrated overlapping and distinct immune features between FAD and FHD. In younger adults, FCEL elicited a more diverse and cytokine-secreting H1 and H3 HA-specific immune response compared to FSD (Fig. 7A, B). FCEL induced a strong activation of CD4^+^ T cells (CD4^+^CD40L^+^IL2^+^, CD4^+^IFN-γ^+^TNF-α^+^) and an expansion of several HA-specific CD4^+^ T cell subsets. In contrast, FSD generated a more limited immune profile, mainly characterized by cTfh1 activation. While some biomarkers were shared across both vaccines, Venn diagrams highlighted the broader and more complex immune response elicited by FCEL. The same profile was observed at D28 post-vaccination in FCEL-vaccinated individuals.

## Discussion

This study provides critical insights into the immunological mechanisms underlying immune responses elicited by four distinct inactivated influenza vaccines in both younger and older adults. Although multiple seasonal influenza vaccines are currently available, there is a lack of confirmed correlates of protection beyond HAI titers and the limited integration of multidimensional immune parameters or clinical assessment.^35–37^ While vaccine immunogenicity does not directly equate to protective efficacy, evaluating coordinated responses among B and T cell compartments provides a more comprehensive understanding of vaccine-induced immunity and can inform the design of next-generation vaccines. While humoral responses were comparable among all vaccinees, by assessing a range of cellular immune parameters, high-dose vaccination consistently outperformed the adjuvanted formulation in older adults, while the cell-based vaccine surpassed the egg-produced standard dose vaccine in younger adults.

All 4 vaccines induced statistically similar HAI titers, with FHD and FCEL yielding the highest seroconversion rates. HAI titers are widely used as a correlate of protection against influenza; however, high HAI titers do not guarantee complete immunity with vaccinated people with high HAI titers may still experience breakthrough infections.^35,36^ HAI assays primarily capture antibody-mediated viral neutralization via HA binding inhibition, overlooking contributions from cellular immunity. Our comprehensive profiling across early and late timepoints post-vaccination revealed distinct immune network signatures among vaccine participants that were not captured by serological assessment alone.

Notably, FHD was the only vaccine to elicit early (day 7) and simultaneous recruitment of ASCs and cTfh1 cells. Consistent with prior studies,^22,23,28,38^ activated cTfh1 cells (ICOS^+^PD-1^+^) were strongly associated with the expansion of ASCs. Interestingly, cTfh was also linked to HAI titers and MBC, suggesting a tightly coordinated T-B cell collaboration and its role in effective humoral and cellular immunity. CD4^+^ Tfh cells are essential for the selection, expansion, and differentiation of MBCs into antibody-secreting plasmablasts and plasma cells. Circulating Tfh cells exhibit many of the same phenotypic and functional attributes as germinal center Tfh cells.^39^ cTfh cells, clonally related to Tfh germinal center-derived counterparts, emerge transiently following influenza vaccination.^40,41^ The superior performance of FHD likely stems from its 4-fold higher HA antigen content. Higher antigen doses in murine models enhance the activation of Tfh cells.^42^ In humans, this approach appears to double the frequency of plasmablasts compared to the standard dose vaccine.^15^ cTfh1 may induce a clonal selection of high-affinity MBCs and drive more robust differentiation of plasmablasts in FHD recipients. In contrast, while the MF59 adjuvant in FAD is known to broaden antigen presentation and enhance innate immune recruitment,^43^ it may not preferentially expand antigen-specific cTfh cells. This could explain the limited capacity of FAD to induce an early plasmablast differentiation compared to FHD. Furthermore, our data suggest that FHD may overcome age-associated impairments in Tfh cells, commonly observed following standard-dose vaccination in older adults.^44^

In brief, cTfh cells play a central role in shaping B cell responses and in this study, HA-specific cTfh and other helper T cell subsets were further evaluated. Although internal viral proteins are often considered dominant T cell targets due to their conservation,^30^ CD4^+^ T cell responses towards the surface HA are crucial for robust antibody production.^29^ Inducing CD4^+^ T cell specificity toward conserved internal epitopes and away from non-conserved HA epitopes significantly reduces the production of HA-specific antibodies.^45^ Vaccines that contain only HA, particularly by excluding internal proteins, generate multifunctional, HA-specific CD4^+^ T cells,^29^ which correlate with both HAI^43^ and microneutralization titers.^46^ FHD and FCEL both induced significantly higher levels of HA-specific CD4^+^ T cells, including double-cytokine-secreting cells and cTfh cells, confirming their capacity to promote both cellular and humoral responses despite their inactivated formulation (not exclusively HA). It is possible that the high amount of antigen in FHD results in less competition with cross-reactive memory CD4^+^ T cells targeting internal epitopes, thereby refocusing CD4^+^ T cells to target HA. Interestingly, the strong HA-specific T cell response induced by FCEL, despite the lack of total cTfh expansion, suggests that its mammalian cell-based production platform may improve antigenic fidelity. By avoiding egg-adaptive mutations that may alter HA epitopes,^39^ FCEL likely preserves more native antigenic sequences, thereby enhancing T cell recognition. In contrast, egg-based vaccines, such as FSD, may introduce epitope alterations that impair T cell and antibody responses.

A hallmark of effective CD4^+^ T cell immunity is the presence of multifunctional effector cells. Both FHD and FCEL induced higher frequencies of HA-specific CD4^+^ T cells producing single and double cytokines. Multifunctional CD4^+^ T cells produce higher amounts of cytokines per cell, express greater levels of co-stimulatory molecules and possess more helper capacity than single-cytokine-producing cells.^47^ Their induction is sensitive to antigen dose^48^ and TCR signaling thresholds,^49^ with higher antigen concentrations favoring double-cytokine-secreting phenotypes that exhibit improved function and proliferative abilities. While MF59 adjuvants enhance antigen presentation, resulting in increased TCR signaling and polyfunctional T cell responses,^50^ the data in this study suggest that in older adults, increasing antigen content may be more effective than adjuvants alone in overcoming immunosenescence and promoting functional CD4^+^ T cell responses. In younger adults, FCEL elicited a strong and multifunctional Th1-skewed response, which is considered critical for protective immunity.^51^ The reduced cellular responses of FSD in this age group may reflect compromised antigenic integrity and potential interference from repeated annual vaccination.^52^

Early immune events may be indicators of long-term vaccine efficacy when correlated with immune memory.^23,32,53^ In this study, HA-specific CD4^+^ T cells elicited by FCEL and FHD on day 7 were associated with serological outcomes at day 28. FHD-induced HA-specific CD4^+^ T cell response was also linked with the rise of MBCs, particularly IgG HA-specific MBCs. This finding supports the notion that high-dose vaccination drives a more robust activation of class-switched memory B cells,^15^ reflecting accelerated recall. Hence, FHD not only promotes acute events, but also that early antigen engagement and CD4 activation, particularly cTfh, are central to the establishment of long-lived immunity. Additionally, the data in this report highlight the role of various immune components (HA-specific AIM^+^ and ICS^+^ CD4^+^ T cells and ASC) as potent predictors of vaccine efficacy and sustained immune responses.

This study has some limitations. First, the B cell assays used recombinant HA from A/Wisconsin/588/2019 (H1N1) (2022–2023 season), which differs from the vaccine strain A/Victoria/4897/2022 (H1N1) (2023–2024 season). However, cross-reactive antibodies were confirmed.^20^ Second, the sample size for matched longitudinal serological and cellular data was limited. Nonetheless, the use of a centralized processing facility and consistent experimental protocols across vaccine arms is a key strength. Additionally, our data capture longitudinal B and T cell responses with a focused analysis on HA specificity, when most approaches are vaccine-specific instead.

In conclusion, all 4 vaccines—FAD, FHD, FCEL, and FSD—induced comparable antibody titers in their respective age groups when assessed conventionally using the HAI method. However, deeper immune profiling revealed that FHD and FCEL elicited an early and more functional HA-specific CD4^+^ T cell response, which was strongly associated with downstream MBC expansion and antibody production. These T cells likely provide essential support for B cell activation, class switching, and the formation of germinal centers. Our findings suggest that the advantage of FHD over FAD in older adults may be due to its higher antigen content, while the efficacy of FCEL over FSD in younger adults likely reflects enhanced antigen quality. These observations should be interpreted with caution, given the limited sample size, and future studies with larger cohorts and clinical endpoints will be required to confirm and extend these results. Nevertheless, our findings underscore the importance of designing vaccines that not only induce robust antibody titers but also activate the cellular networks necessary for durable and protective immunity, especially in vulnerable populations, such as the elderly. Synergistic activation of multiple immune compartments, including ASCs, cTfh cells, MBCs, and HA-specific effector CD4^+^ T cells, should be considered to establish immunological memory.

## Supplementary Material

Supplementary files word

Supplementary files pdf

Supplementary material

Supplementary material is available at *The Journal of Immunology* online.

## Figures and Tables

**Table 1.** Study population characteristics.

	Vaccination group							
	Older adults				Younger adults			
	Fluad (*n* = 13)		Fluzone-High-Dose (*n* = 13)		Fluzone-Standard-Dose (*n* = 13)		Flucelvax (*n* = 13)	

*Age, n* (%)	GM = 72		GM = 73		GM = 44		GM = 40	
25–34	…	…	…	…	3	(23%)	5	(38%)
35–44	…	…	…	…	2	(15%)	1	(8%)
45–60	…	…	…	…	8	(62%)	7	(54%)
65–74	8	(62%)	8	(62%)	…	…	…	–
75–84	4	(31%)	4	(31%)	…	…	…	…
85+	1	(8%)	1	(8%)	…	…	…	…
*Gender, n* (%)								
Male	4	(31%)	5	(38%)	3	(23%)	1	(8%)
Female	9	(69%)	8	(62%)	10	(77%)	12	(92%)
*Race or ethnicity, n* (%)								
White: Non-Hispanic/Latino	12	(92%)	13	(100%)	11	(85%)	6	(46%)
White: Hispanic/Latino	0	(0%)	0	(0%)	0	(0%)	5	(38%)
Black	1	(8%)	0	(0%)	2	(15%)	2	(15%)
Other	0	(0%)	0	(0%)	0	(0%)	0	(0%)

## Data Availability

The original data presented in the study are made publicly available via by the National Institutes of Health ImmPort online database.
