# Supplementary material for: Comparative analysis of cellular immune responses to four seasonal inactivated influenza vaccines in younger and older adults
Source: J Immunol. 2025 Oct 30;215(2):vkaf286. doi: 10.1093/jimmun/vkaf286 (PMC13011262; doi:10.1093/jimmun/vkaf286)
Supplement: vkaf286_Supplementary_Data [file vkaf286_supplementary_data.docx]

**Figure S1. Representative gating strategy. (a)** ASC (also known as plasmablast) and total cTfh analysis from PBMC collected before and 7 days post inactivated influenza virus vaccination, **(b)** HA-specific MBC analysis from PBMC collected before and 28 and 90 days post inactivated influenza virus vaccination, and **(c)** HA-specific AIM+ and ICS CD4^+^ T cell analysis from PBMC collected before and 7, 28 and 90 days post inactivated influenza virus vaccination. AIM: activation-induced marker, ICS: intracellular cytokine staining, MBC: memory B cells, ASC: antibody-secreting cells, cTfh: circulating T follicular helper cells,

**Table SI. FACS reagents and concentrations.**

| a | **B CELL (EARLY RESPONSE - D0, D7)** | | | | | | |
| --- | --- | --- | --- | --- | --- | --- | --- |
|  | Detector | Antibody | Fluorochrome | ul/well | Cat. Number | Vendor | Clone |
|  | B2 | HLADR | FITC | 1.00 | 555811 | BD Pharmigen | G46-6 |
|  | B3 | CD8 | SB550 | 0.20 | 344760 | Biolegend | SK1 |
|  | B9 | CXCR3 | BB700 | 4.00 | 566532 | BD Horizon | 1C6/CXCR3 |
|  | R4 | CD3 | AF700 | 1.00 | 317340 | Biolegend | OKT3 |
|  | R7 | CD16 (dump) | APC-CY7 | 0.20 | 302018 | Biolegend | 3G8 |
|  | R7 | CD14 (dump) | APC-CY7 | 0.25 | 301820 | Biolegend | M5E2 |
|  | R7 | LD | NIR | 0.03 | L10119 | Invitrogen |  |
|  | V01 | CD19 | BV421 | 1.00 | 562441 | Biolegend | HIB19 |
|  | V11 | ICOS | BV650 | 4.00 | 563832 | BD Horizon | DX29 |
|  | V13 | CXCR5 | BV711 | 4.00 | 356934 | Biolegend | J252D4 |
|  | V15 | CD4 | BV785 | 0.25 | 300554 | Biolegend | RPA-T4 |
|  | YG1 | CCR6 | PE | 1.00 | 353410 | Biolegend | G034E3 |
|  | YG3 | CD38 | PE-Dazzle | 1.00 | 303538 | Biolegend | HIT2 |
|  | YG5 | PD1 | PE-CY5 | 2.00 | 329972 | Biolegend | EH12.2H7 |
|  | YG9 | CD27 | Pe-CY7 | 0.50 | 560609 | BD Pharmigen | M-T271 |

| b | **B CELL (MEMORY RESPONSE - D0, D28, D90)** | | | | | | |
| --- | --- | --- | --- | --- | --- | --- | --- |
|  | Detector | Antibody | Fluorochrome | ul/well | Cat. Number | Vendor | Clone |
|  | B10 | IgA | PerCP-Vio700 | 2.00 | 130-107-050 | Miltenyi Biotec | IS11-21E11 |
|  | R1 | H3 | APC |  |  | *In house* |  |
|  | R4 | CD38 | AF700 | 4.00 | 303524 | Biolegend | HIT2 |
|  | R7 | CD10 (dump) | APC-CY7 | 0.50 | 312212 | Biolegend | HI10a |
|  | R7 | CD16 (dump) | APC-CY7 | 0.20 | 302018 | Biolegend | 3G8 |
|  | R7 | CD14 (dump) | APC-CY7 | 0.25 | 301820 | Biolegend | M5E2 |
|  | R7 | CD3 (dump) | APC-CY7 | 0.20 | 317342 | Biolegend | OKT3 |
|  | R7 | LD | NIR | 0.03 | L10119 | Invitrogen |  |
|  | R7 | SA-FREE | APC-CY7 | 10 ul of '1:200 | 405208 | Biolegend |  |
|  | V01 | IgD | BV421 | 0.50 | 348226 | Biolegend | IA6-2 |
|  | V05 | CD21 | BV480 | 0.50 | 746613 | BD OptiBuild | B-ly4 |
|  | V07 | IgG | BV510 | 5.00 | 563247 | BD Horizon | G18-145 |
|  | V10 | CD20 | BV605 | 2.00 | 302334 | Biolegend | 2H7 |
|  | V14 | CD19 | BV750 | 0.60 | 302262 | Biolegend | HIB19 |
|  | V15 | IgM | BV786 | 1.00 | 740998 | BD OptiBuild | G20-127 |
|  | YG1 | H1 | PE |  |  | *In house* |  |
|  | YG9 | CD27 | Pe-CY7 | 0.50 | 560609 | BD Pharmigen | M-T271 |

| c | **T CELL (AIM + ICS - D0, D7, D28, D90)** | | | | | | | |
| --- | --- | --- | --- | --- | --- | --- | --- | --- |
|  |  | Detector | Antibody | Fluorochrome | ul/well | Cat. Number | Vendor | Clone |
|  | INTRA | B03 | GZB | AF532 | 3 | 58-8896-42 | Invitrogen | N4TL33 |
|  | INTRA | B10 | IL2 | PerCP-eFluor 710 | 1 | 46-7029-42 | Invitrogen | MQ1-17H12 |
|  | INTRA | R1 | IL4 | APC | 2 | 500812 | Biolegend | MP4-25D2 |
|  | INTRA | V01 | IL10 | BV421 | 2 | 501422 | Biolegend | JES3-9D7 |
|  | INTRA | V07 | CD69 | BV510 | 2 | 310936 | Biolegend | FN50 |
|  | INTRA | V14 | CD137 | BV750 | 1 | 309844 | Biolegend | 4B4-1 |
|  | INTRA | V15 | TNFa | BV785 | 1 | 502948 | Biolegend | Mab11 |
|  | INTRA | YG3 | IFNG | PE-CF594 | 0.5 | 562392 | BD Horizon | B27 |
|  | INTRA | B04 | CD3 | SB574 | 1 | 300487 | Biolegend | UCHT1 |
|  | SURF | B08 | CD45RA | PerCP | 2 | 304156 | Biolegend | HI100 |
|  | SURF | B09 | CXCR3 | BB700 | 4 | 566532 | BD Horizon | 1C6/CXCR3 |
|  | SURF | R4 | CCR7 | APC-R700 | 2 | 566766 | BD Horizon | 2-L1-A |
|  | SURF | R7 | CD14 | APCCY7 | 0.25 | 301820 | Biolegend | M5E2 |
|  | SURF | R7 | CD16 | APCCY7 | 0.20 | 302018 | Biolegend | 3G8 |
|  | SURF | R7 | CD19 | APC FIRE 750 | 0.2 | 302258 | Biolegend | HIB19 |
|  | SURF | R7 | LD NIR |  | 0.03 | L10119 | Invitrogen |  |
|  | SURF | V03 | CD4 | PB | 1 | 317429 | Biolegend | OKT4 |
|  | SURF | V10 | TIGIT | BV605 | 4 | 372712 | Biolegend | A15153G |
|  | SURF | V11 | ICOS | BV650 | 4 | 563832 | BD Horizon | DX29 |
|  | SURF | V13 | CXCR5 | BV711 | 4 | 356934 | Biolegend | J252D4 |
|  | SURF | YG1 | CCR6 | PE | 1 | 353410 | Biolegend | G034E3 |
|  | SURF | YG5 | PD1 | PE-CY5 | 2 | 329972 | Biolegend | EH12.2H7 |
|  | SURF | YG7 | CD8 | PE CY5.5 | 0.4 | 35-0088-42 | Invitrogen | RPA-T8 |
|  | SURF | YG9 | CD40L | Pe-Vio770 | 1 | 130-113-608 | Miltenyi Biotec | 5C8 |

**(a)** B cell early **(**ASC and total cTfh analysis) from PBMC collected before and 7 days post inactivated influenza virus vaccination, **(b)** MBC (HA-specific B memory cell, Ig Isotypes and activation phenotypes) from PBMC collected before and 28 and 90 days post inactivated influenza virus vaccination, and **(c)** T cell **(**HA-specific AIM^+^ and ICS^+^ CD4^+^ T cell analysis) from PBMC collected before and 7, 28 and 90 days post inactivated influenza virus vaccination. INTRA: intracellular staining, SURF: surface staining, AIM: activation-induced marker, ICS: intracellular cytokine staining, MBC: memory B cells, ASC: antibody-secreting cells, cTfh: circulating T follicular helper cells.

**Figure S2. Fold change (d7/d0) in activated cTfh1 frequency post vaccination.** The vaccine groups are color-coded (n=13 per group). The data are represented as median and IQR. Data were analyzed for statistical significance using the Mann-Whitney test. Only significant data are shown, as indicated on the graphs. See Figure S1A for the complete gating strategy. ASC: antibody-secreting cells, cTfh1 type-1 circulating T follicular helper. FAD: Fluad, FHD: Fluzone High-Dose, FSD: Fluzone Standard-Dose, and FCEL: Flucelvax**.**

**Figure S3. Correlation analysis between the rise of HA-MBCs and HAI titers at day 28 in FHD recipients.** HA-specific CD4^+^ T cells were measured as a percentage of AIM^+^ (CD40L^+^CD69^+^) CD4^+^ T cells following stimulation of PBMCs with a peptide pool spanning the HA protein from H1N1 and H3N2. HA-specific functional CD4^+^ T cells were quantified as the percentage of CD40L^+^ secreted effector cells producing IFN-γ, TNF-α, and IL-2 specific to H1N1 and H3N2. Data are presented as background-subtracted paired with unstimulated samples. Statistical significance was assessed using Spearman’s correlation. Colored values refer to the group legend within the graph. Significant data is underlined, while trending data is bolded. See Figure S1B for the complete gating strategy. ASC: antibody-secreting cells, AIM: activation-induced marker, ICS: intracellular cytokine staining, FHD: Fluzone High-Dose, MBC: memory B cells.
