# Supplementary material for: Comparative analysis of cellular immune responses to four seasonal inactivated influenza vaccines in younger and older adults
Source: J Immunol. Author manuscript; Available in PMC 2026 Mar 24. (PMC13011262; doi:10.1093/jimmun/vkaf286)
Supplement: Supplementary file 1 [file NIHMS2149388-supplement-Supplementary_files_pdf.pdf]

a

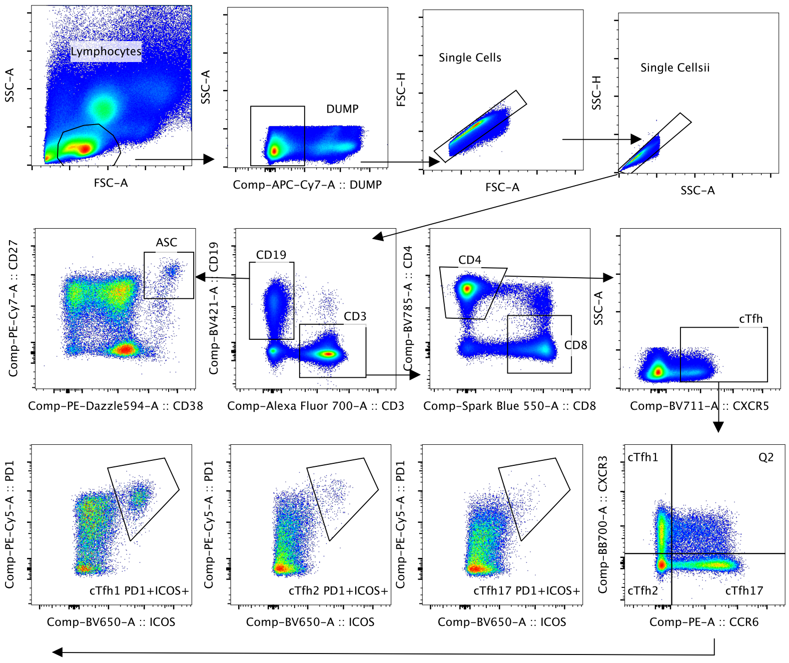

Lymphocytes
SSC-A
FSC-A
DUMP
Comp-APC-Cy7-A :: DUMP
Single Cells
FSC-H
FSC-A
Single Cellsii
SSC-H
SSC-A
ASC
Comp-PE-Cy7-A :: CD27
Comp-PE-Dazzle594-A :: CD38
CD19
CD3
Comp-BV421-A :: CD19
Comp-Alexa Fluor 700-A :: CD3
CD4
CD8
Comp-BV785-A :: CD4
Comp-Spark Blue 550-A :: CD8
cTfh
Comp-BV711-A :: CXCR5
Comp-PE-Cy5-A :: PD1
cTfh1 PD1+ICOS+
Comp-BV650-A :: ICOS
cTfh2 PD1+ICOS+
cTfh17 PD1+ICOS+
cTfh1
Q2
cTfh2
cTfh17
Comp-BB700-A :: CXCR3
Comp-PE-A :: CCR6

b

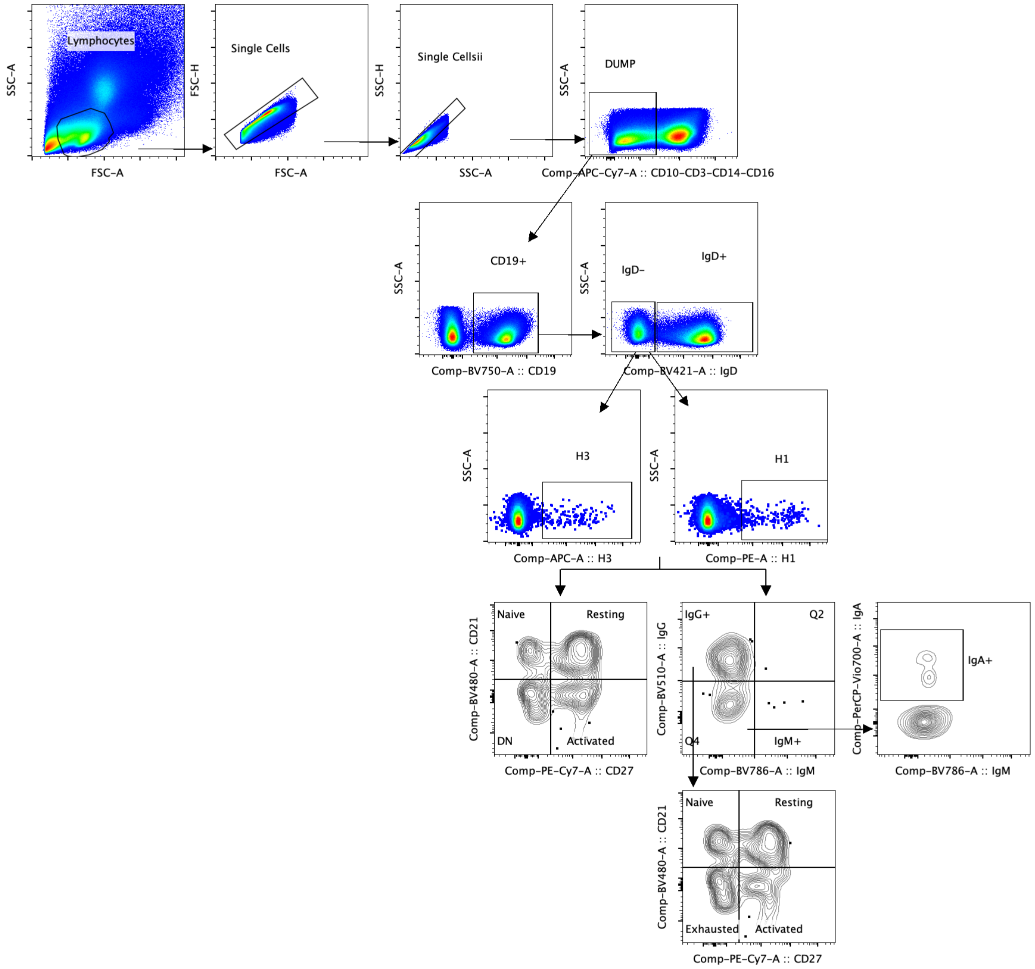

Lymphocytes
SSC-A
FSC-A
Single Cells
FSC-H
Single Cellsii
SSC-H
DUMP
Comp-APC-Cy7-A :: CD10-CD3-CD14-CD16
CD19+
Comp-BV750-A :: CD19
IgD-
IgD+
Comp-BV421-A :: IgD
H3
Comp-APC-A :: H3
H1
Comp-PE-A :: H1
Naive
Resting
DN
Activated
Comp-BV480-A :: CD21
Comp-PE-Cy7-A :: CD27
IgG+
Q2
Q4
IgM+
Comp-BV510-A :: IgG
Comp-BV786-A :: IgM
IgA+
Comp-PerCP-Vio700-A :: IgA
Exhausted

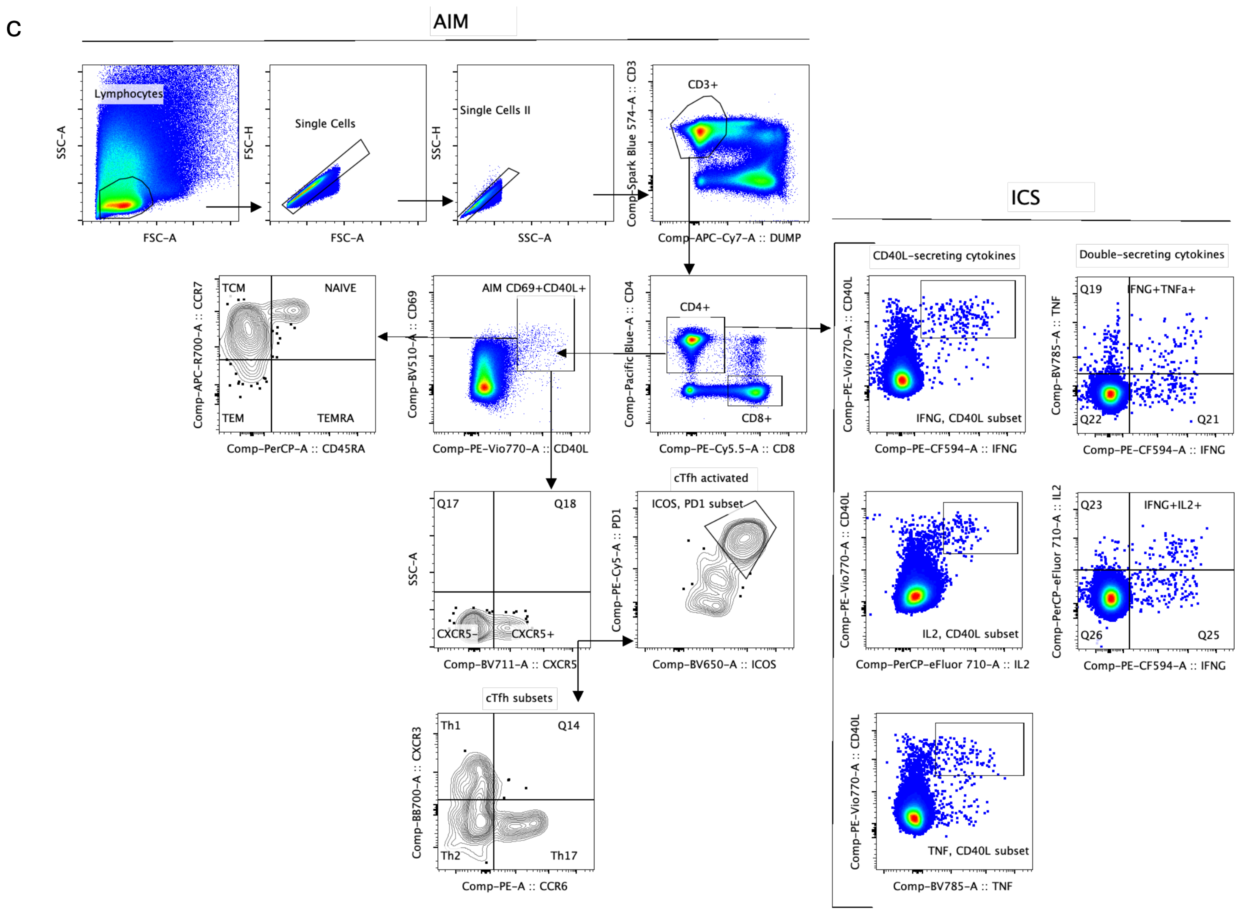

Figure S1. Representative gating strategy. (a) ASC (also known as plasmablast) and total cTfh analysis from PBMC collected before and 7 days post inactivated influenza virus vaccination, **(b)** HA-specific MBC analysis from PBMC collected before and 28 and 90 days post inactivated influenza virus vaccination, and **(c)** HA-specific AIM+ and ICS CD4^{+} T cell analysis from PBMC collected before and 7, 28 and 90 days post inactivated influenza virus vaccination. AIM: activation-induced marker, ICS: intracellular cytokine staining, MBC: memory B cells, ASC: antibody-secreting cells, cTfh: circulating T follicular helper cells,

Table SI. FACS reagents and concentrations.

a

B CELL (EARLY RESPONSE - D0, D7)						
Detector	Antibody	Fluorochrome	ul/well	Cat. Number	Vendor	Clone
B2	HLADR	FITC	1.00	555811	BD Pharmigen	G46-6
B3	CD8	SB550	0.20	344760	Biolegend	SK1
B9	CXCR3	BB700	4.00	566532	BD Horizon	1C6/CXCR3
R4	CD3	AF700	1.00	317340	Biolegend	OKT3
R7	CD16 (dump)	APC-CY7	0.20	302018	Biolegend	3G8
R7	CD14 (dump)	APC-CY7	0.25	301820	Biolegend	M5E2
R7	LD	NIR	0.03	L10119	Invitrogen	
V01	CD19	BV421	1.00	562441	Biolegend	HIB19
V11	ICOS	BV650	4.00	563832	BD Horizon	DX29
V13	CXCR5	BV711	4.00	356934	Biolegend	J252D4
V15	CD4	BV785	0.25	300554	Biolegend	RPA-T4
YG1	CCR6	PE	1.00	353410	Biolegend	G034E3
YG3	CD38	PE-Dazzle	1.00	303538	Biolegend	HIT2
YG5	PD1	PE-CY5	2.00	329972	Biolegend	EH12.2H7
YG9	CD27	Pe-CY7	0.50	560609	BD Pharmigen	M-T271

b

B CELL (MEMORY RESPONSE - D0, D28, D90)						
Detector	Antibody	Fluorochrome	ul/well	Cat. Number	Vendor	Clone
B10	IgA	PerCP-Vio700	2.00	130-107-050	Miltenyi Biotec	IS11-21E11
R1	H3	APC			*In house*	
R4	CD38	AF700	4.00	303524	Biolegend	HIT2
R7	CD10 (dump)	APC-CY7	0.50	312212	Biolegend	HI10a
R7	CD16 (dump)	APC-CY7	0.20	302018	Biolegend	3G8
R7	CD14 (dump)	APC-CY7	0.25	301820	Biolegend	M5E2
R7	CD3 (dump)	APC-CY7	0.20	317342	Biolegend	OKT3
R7	LD	NIR	0.03	L10119	Invitrogen	
R7	SA-FREE	APC-CY7	10 ul of '1:200	405208	Biolegend	
V01	IgD	BV421	0.50	348226	Biolegend	IA6-2
V05	CD21	BV480	0.50	746613	BD OptiBuild	B-ly4
V07	IgG	BV510	5.00	563247	BD Horizon	G18-145
V10	CD20	BV605	2.00	302334	Biolegend	2H7
V14	CD19	BV750	0.60	302262	Biolegend	HIB19
V15	IgM	BV786	1.00	740998	BD OptiBuild	G20-127
YG1	H1	PE			*In house*	

YG9	CD27	Pe-CY7	0.50	560609	BD Pharmigen	M-T271

c

T CELL (AIM + ICS - D0, D7, D28, D90)							
	Detector	Antibody	Fluorochrome	ul/well	Cat. Number	Vendor	Clone
INTRA	B03	GZB	AF532	3	58-8896-42	Invitrogen	N4TL33
INTRA	B10	IL2	PerCP-eFluor 710	1	46-7029-42	Invitrogen	MQ1-17H12
INTRA	R1	IL4	APC	2	500812	Biolegend	MP4-25D2
INTRA	V01	IL10	BV421	2	501422	Biolegend	JES3-9D7
INTRA	V07	CD69	BV510	2	310936	Biolegend	FN50
INTRA	V14	CD137	BV750	1	309844	Biolegend	4B4-1
INTRA	V15	TNFa	BV785	1	502948	Biolegend	Mab11
INTRA	YG3	IFNG	PE-CF594	0.5	562392	BD Horizon	B27
INTRA	B04	CD3	SB574	1	300487	Biolegend	UCHT1
SURF	B08	CD45RA	PerCP	2	304156	Biolegend	HI100
SURF	B09	CXCR3	BB700	4	566532	BD Horizon	1C6/CXCR3
SURF	R4	CCR7	APC-R700	2	566766	BD Horizon	2-L1-A
SURF	R7	CD14	APCCY7	0.25	301820	Biolegend	M5E2
SURF	R7	CD16	APCCY7	0.20	302018	Biolegend	3G8
SURF	R7	CD19	APC FIRE 750	0.2	302258	Biolegend	HIB19
SURF	R7	LD NIR		0.03	L10119	Invitrogen	
SURF	V03	CD4	PB	1	317429	Biolegend	OKT4
SURF	V10	TIGIT	BV605	4	372712	Biolegend	A15153G
SURF	V11	ICOS	BV650	4	563832	BD Horizon	DX29
SURF	V13	CXCR5	BV711	4	356934	Biolegend	J252D4
SURF	YG1	CCR6	PE	1	353410	Biolegend	G034E3
SURF	YG5	PD1	PE-CY5	2	329972	Biolegend	EH12.2H7
SURF	YG7	CD8	PE CY5.5	0.4	35-0088-42	Invitrogen	RPA-T8
SURF	YG9	CD40L	Pe-Vio770	1	130-113-608	Miltenyi Biotec	5C8

(a) B cell early **(**ASC and total cTfh analysis) from PBMC collected before and 7 days post inactivated influenza virus vaccination, **(b)** MBC (HA-specific B memory cell, Ig Isotypes and activation phenotypes) from PBMC collected before and 28 and 90 days post inactivated influenza virus vaccination, and **(c)** T cell **(**HA-specific AIM$^+$ and ICS$^+$ CD4$^+$ T cell analysis) from PBMC collected before and 7, 28 and 90 days post inactivated influenza virus vaccination. INTRA: intracellular staining, SURF: surface staining, AIM: activation-induced marker, ICS: intracellular cytokine staining, MBC: memory B cells, ASC: antibody-secreting cells, cTfh: circulating T follicular helper cells.

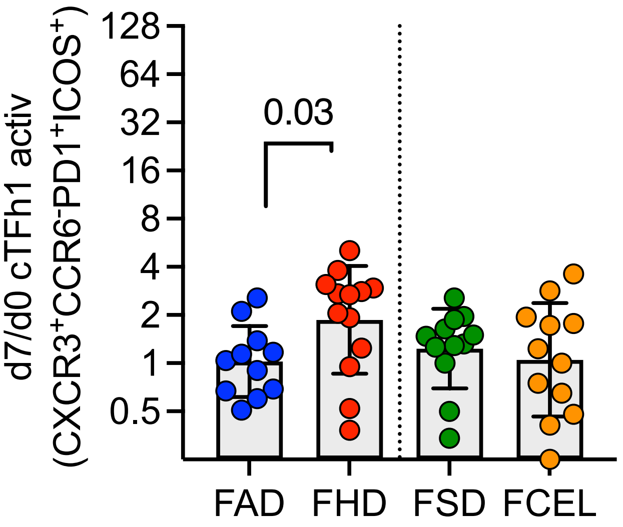

Figure S2. Fold change (d7/d0) in activated cTfh1 frequency post vaccination. The vaccine groups are color-coded (n=13 per group). The data are represented as median and IQR. Data were analyzed for statistical significance using the Mann-Whitney test. Only significant data are shown, as indicated on the graphs. See Figure S1A for the complete gating strategy. ASC: antibody-secreting cells, cTfh1 type-1 circulating T follicular helper. FAD: Fluad, FHD: Fluzone High-Dose, FSD: Fluzone Standard-Dose, and FCEL: Flucelvax**.**

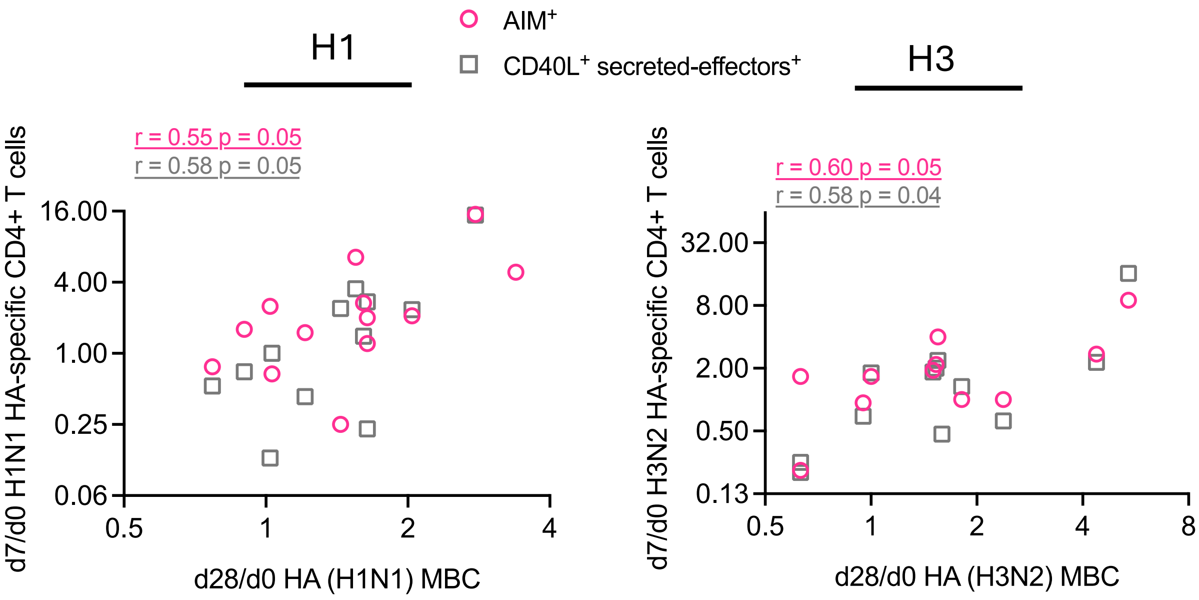

Figure S3. Correlation analysis between the rise of HA-MBCs and HAI titers at day 28 in FHD recipients. HA-specific $CD4^+$ T cells were measured as a percentage of AIM^+ ($CD40L^+CD69^+$) $CD4^+$ T cells following stimulation of PBMCs with a peptide pool spanning the HA protein from H1N1 and H3N2. HA-specific functional $CD4^+$ T cells were quantified as the percentage of $CD40L^+$ secreted effector cells producing IFN-γ, TNF-α, and IL-2 specific to H1N1 and H3N2. Data are presented as background-subtracted paired with unstimulated samples. Statistical significance was assessed using Spearman's correlation. Colored values refer to the group legend within the graph. Significant data is underlined, while trending data is bolded. See Figure S1B for the complete gating strategy. ASC: antibody-secreting cells, AIM: activation-induced marker, ICS: intracellular cytokine staining, FHD: Fluzone High-Dose, MBC: memory B cells.
